# Phage cocktail containing *Podoviridae* and *Myoviridae* bacteriophages inhibits the growth of *Pectobacterium* spp. under *in vitro* and *in vivo* conditions

**DOI:** 10.1371/journal.pone.0230842

**Published:** 2020-04-02

**Authors:** Maja A. Zaczek-Moczydłowska, Gillian K. Young, James Trudgett, Cali Plahe, Colin C. Fleming, Katrina Campbell, Richard O’ Hanlon

**Affiliations:** 1 Institute for Global Food Security, School of Biological Sciences, Queen's University, Belfast, Northern Ireland, United Kingdom; 2 Sustainable Agri-Food Sciences Division, Agri-Food and Biosciences Institute, Belfast, Northern Ireland, United Kingdom; 3 Veterinary Sciences Division, Agri-Food and Biosciences Institute, Stormont, Belfast, Northern Ireland, United Kingdom; 4 School of Biological Sciences, Queen's University, Belfast, Northern Ireland, United Kingdom; Dong-A University, REPUBLIC OF KOREA

## Abstract

Globally, there is a high economic burden caused by pre- and post–harvest losses in vegetables, fruits and ornamentals due to soft rot diseases. At present, the control methods for these diseases are limited, but there is some promise in developing biological control products for use in Integrated Pest Management. This study sought to formulate a phage cocktail which would be effective against soft rot *Pectobacteriaceae* species affecting potato (*Solanum tuberosum* L.), with potential methods of application in agricultural systems, including vacuum–infiltration and soil drench, also tested. Six bacteriophages were isolated and characterized using transmission electron microscopy, and tested against a range of *Pectobacterium* species that cause soft rot/blackleg of potato. Isolated bacteriophages of the family *Podoviridae* and *Myoviridae* were able to control isolates of the *Pectobacterium* species: *Pectobacterium atrosepticum* and *Pectobacterium carotovorum* subsp. *carotovorum*. Genomic analysis of three *Podoviridae* phages did not indicate host genes transcripts or proteins encoding toxin or antibiotic resistance genes. These bacteriophages were formulated as a phage cocktail and further experiments showed high activity *in vitro* and *in vivo* to suppress *Pectobacterium* growth, potentially indicating their efficacy in formulation as a microbial pest control agent to use *in planta*.

## Introduction

*Pectobacteriaceae* species cause pre–and post–harvest losses to potato production (*S*. *tuberosum* L.) world–wide, which is one of the most intensively cultivated food crops globally [1, 2, 3]. Recent studies have highlighted the potential of using lytic bacteriophages as a microbial pest control agent (MPCA) to control plant diseases caused by soft rot *Pectobacteriaceae* (SRP) [4, 5], with one product being commercially introduced in the UK, to protect potatoes in storage against soft rot as a pre–packing wash solution [6].

Research in plant, animal and human health has indicated several factors which may influence the success of phage therapy [7, 8, 9], and effective application in agriculture under natural environmental conditions might be difficult to achieve [10]. These factors include levels of bacterial populations and concentrations of bacteriophage virion at the application site [11], temperature, pH of the rhizosphere, moisture, organic content of the soil [12], or the possibility of phage–resistant bacterial mutants, e.g. occurring due to modifications in phage receptors or the development of several bacterial defence mechanisms [10]. To counteract this threat, multiple bacteriophage formulations have been recently tested to control losses caused by SRP [4, 5, 13].

Several research studies have characterized bacteriophages with the aim of formulating a MPCA against SRP *in vitro*, to target emerging pathogen species in several countries [14]. In previous studies, characterization of potential candidates for phage-based MPCA was carried out according to morphotype, host range, lytic activity, and genome characterization [15, 16, 17, 13, 18, 19, 20, 5, 21, 22]. Though not all studies provided evidence of bacteriophage efficacy in bioassays and/or field trials, two studies provided details of semi–*in planta* experiments on potatoes involving a multiple bacteriophage mixture. Czajkowski and co–workers [23] identified a protective effect of two bacteriophages (ϕPD10.3 and ϕPD23.1) combined together in two experimental bioassays, on potato tuber slices and in whole tuber assays, against a mix of *Pectobacteriaceae* spp. including *Dickeya solani*, *P*. *carotovorum* subsp. *carotovorum* and *Pectobacterium wasabiae*. These two bacteriophages significantly reduced potato tuber tissue maceration by over 80% of the control potato slices and by over 95% of the control whole tubers [23]. Three bacteriophages (φCB1, φCB3, and φCB4) in a mixture were able to significantly suppress bacterial (*P*. *atrosepticum)* growth in a whole tuber assay [5]. Two further studies revealed bacterial inhibition by selected bacteriophages in semi–*in planta* assays on potato tubers; however, these bacteriophages were tested as monophage formulation and against a narrow range of SRP [24, 21]. Smolarska and co–workers [21] provided details of *Pectobacterium parmentieri* suppression growth in a potato tuber assay after inoculation with two bacteriophages: φA38 and φA41. These bacteriophages were able to reduce potato tuber tissue maceration to 40–50% of that observed in the positive control [21]. Lee and co-workers [24] reported a decrease of rotting symptoms using isolated bacteriophages against *P*. *carotovorum* subsp. *carotovorum* [24].

To date, limited success has been achieved in formulating a bacteriophage based MPCA to control SRP (e.g. *Dickeya* sp.) under field conditions [16]. Adriaennsens and co–workers [16] reported greenhouse control of bacterial soft rot in potato tubers with two bacteriophages named Limestone 1 and Limestone 2. These bacteriophages were also tested under field conditions, though they did not show significant differences in terms of emergence and suppression of soft rot.

The aim of this work was to isolate and characterize bacteriophages for their efficacy in controlling bacterial soft rot caused by *P*. *atrosepticum* and *P*. *carotovorum* subsp. *carotovorum in vitro* and *in vivo*.

## Materials and methods

"Field work has been performed under permission of Agri-Food and Biosciences Institute, College of Agriculture, Food and Rural Enterprise and Department of Agriculture, Environment and Rural Affairs in Northern Ireland".

### Bacterial isolates and media

Reference *Pectobacterium* strains used in this study (S1 Table) were characterized previously [25]. Isolates were stored at 4°C for long-term storage. Prior to use, *Pectobacterium* strains grown at 25°C for 24–48 h were purified twice on nutrient agar (NA) (CM0003, Oxoid). For liquid applications of bacteria, pure bacterial colonies were harvested and inoculated in nutrient broth (NB) containing peptone (1g, Sigma Aldrich), yeast extract (0.5g, Oxoid), NaCl (0.25g, Fisher Scientific), K_2_HPO_4_ (0.8g, Fisher Scientific) per 100 ml, for 12–24 h at 25°C with 200 rpm agitation and adjusted to *ca*. 10^8^ cfu ml^-1^ in NB (approx. OD_600nm_ = 0.2). Prior to performing semi–*in planta* experiments, a pathogenicity test was performed by inoculating a suspension of (*P*. *atrosepticum* and *P*. *carotovorum* subsp. *carotovorum*) *ca*. 10^8^ cfu ml^-1^ of a 12–24 h day old culture on sterilised potato half–tubers.

### Isolation and purification of bacteriophages

#### Filtration of processing water samples

Bacteriophages were isolated from the potato processing water samples provided by Department of Agriculture and Rural Affairs (DAERA) by filtration using Filtration unit Stericup^™^ Millipore Express TM® Plus 0.22 μm (Merck): Steritop^™^ filter and a filter receiver flask, connected to a general purpose vacuum pump (KNF Neuberger, ultimate vacuum 100 Mbar, flow rate 15 l min^-1^). A volume of 40 ml of processing water sample was poured into the Steritop^™^ filter to obtain pure bacteriophage filtrate in the receiver flask.

#### Bacteriophage enrichment

For enrichment of bacteriophages, a volume of 5 ml of sterile 10 x NB containing peptone (20 g, Sigma Aldrich), yeast extract (10 g, Oxoid), NaCl (5 g, Fisher Scientific) and K_2_HPO_4_ (16 g, Fisher Scientific) per 200 ml was added to the Stericup^™^ Millipore Express TM® Plus filter receiver flask (Merck) containing the phage-cell filtrate, followed by equal volumes (2.5 ml) of *P*. *carotovorum* and *P*. *atrosepticum* (S1 Table) of liquid bacterial cultures at a cell concentration of *ca*. 10^8^ cfu ml^-1^ (OD_600nm_ = 0.2). The resulting solution (bacteriophage and bacteria) was incubated at 25°C with 200 rpm agitation for 12–24 h. Following that, an aliquot of 10 ml of the solution was transferred into a centrifuge tube and centrifuged at (2000 rpm, 5°C) for 5 min. The supernatant which contained bacteriophages was filtered using a 10 ml syringe barrel fitted with a 0.22 μm filter Millex® GV filter unit (Merck). This bacteriophage filtrate was stored at 4°C until use. The 100 μl of filtrate was added to 900 μl of sterile phosphate-buffered saline (PBS) buffer containing NaCl (1.6g, Sigma Aldrich), KCl (0.04g, Fisher Scientific), K_2_HPO_4_ (0.22g, Sigma Aldrich) and KH_2_PO_4_ (0.04g, Fisher Scientific) per 100 ml, pH 7.4. Five–fold serial dilutions were made in PBS buffer pH 7.4 (Neat, 10^−1^, 10^−2^, 10^−3^, 10^−4^) and subjected to plaque formation using the double layer agar method.

#### Plaque formation using double layer agar method

Cell–phage mix was combined with equal volumes (250 μl) of each liquid bacterial culture of *P*. *carotovorum* and *P*. *atrosepticum* (S1 Table) and 100 μl of each bacteriophage in a five–fold dilution and then incubated at 25°C for 20 min to allow the phage to adsorb to the bacteria. The 3–5 ml of top agarose (TA) (37°C) containing peptone (10g, Sigma Aldrich), yeast extract (5g, Oxoid), NaCl (2.5g, Fisher Scientific), K_2_HPO_4_ (8g, Fisher Scientific) and low gelling agarose (7.5g, Sigma Aldrich) per litre was added to a 30 ml Sterilin® universal container (Thermo Fisher Scientific). The container was capped quickly and mixed gently. The mixture was immediately poured onto the NA plates (CM0003, Oxoid) and left until the agarose solidified, and then incubated at 25°C for 24 h.

#### Purification of bacteriophages

Bacteriophages were harvested by picking plaques obtained on NA plates, using sterile pipette tips and eluting in 900 μl of PBS buffer pH 7.4. Five–fold dilutions were subsequently made and replated. This step was repeated 3–5 times until clear plaque morphology was obtained.

#### Bacteriophage lysate

Double layer plates with pure phage plaques were subsequently re–suspended by adding 4 ml of PBS buffer, pH 7.4 to obtain bacteriophage lysate. After 3 h, harvested lysate was filter–sterilized using a 10 ml syringe barrel fitted (Thermo) with a 0.22 μm filter Millex® GV filter unit (Merck) and maintained in a 30 ml Sterilin® universal container (Thermo Fisher Scientific) at 4°C.

#### Titration of bacteriophages lysate

To determine the titre concentration, a volume of 10 μl of lysate was added to 990 μl of PBS buffer pH 7.4. Ten–fold serial dilutions were made in PBS buffer pH 7.4 and subjected to plaque formation for 24 h using the double layer agar method. The concentration of each bacteriophage lysate was expressed as plaque forming units (pfu ml^-1^).

### Determination of bacteriophage morphotype diversity using transmission electron microscopy (TEM)

To obtain a high titre of bacteriophage, 1.5 ml of bacteriophage lysate was added to 11 ml of PBS and 7 ml of bacteria inoculated in NB for growth for 24 h at 25°C (200 rpm) with agitation. The mixture was filter sterilised using a 0.22 μm filter Millex® GV filter unit (Merck). The bacteriophage lysate (*ca*. 10^8^–10^14^ pfu ml^-1^) was concentrated and purified by centrifugation with modifications to the method previously described [26]. The modification involved centrifugation at 30 000 x g, 4°C, and washing with 500 μl 1 M of ammonium acetate (pH 7.4). The pellet (10 μl) taken from the bottom of the Eppendorf tube was placed on a glass plate, and a copper grid was placed on the sample for 15 min to adsorb. The copper grids were then placed in negative stain (4% ammonium molybdate) (Sigma Aldrich) for 2 min. The excess of liquid was removed using Whatman® paper. The grids were dried for 5 min, then observed at 80 kv using transmission electron microscope JEM–1400 TEM (JEOL, USA).

### General characteristics of bacteriophages

#### Screening bacteriophages against isolated SRP

Six bacteriophages, isolated from potato processing water, were tested for virulence using spotting, and an overlay assay against 18 bacterial isolates. Bacteria that originated from Northern Ireland were isolated in the years 2014–2016 from macerated potato tissue (S1 Table).

For the spotting assay, the selected *Pectobacterium* strains were cultured in NB, at 25°C for 12–24 hours with agitation (200 rpm). A total of 250 μl of the resulting liquid bacterial culture was inoculated into TA (5 ml, 37°C). After gentle vortexing of this mixture, it was poured into prepared NA (CM0003, Oxoid) plates and allowed to solidify at room temperature for 30 min to produce bacterial lawns. Then, 20 μl of phage lysate (*ca*. 10^8^ pfu ml^-1^) was spotted using a pipette onto the TA layer, and the plates were left to dry at room temperature for 30 min. These plates were incubated overnight at 25°C and inspected the next day for inhibition zones.

For the overlay assay, phage stock dilutions (100 μl) were mixed with 250 μl of each *P*. *atrosepticum* and *P*. *carotovorum* subsp. *carotovorum* cultures (S1 Table) and then incubated for 20 min at 25°C. The mixture was combined with TA (5 ml, 37°C), then poured into NA (CM003, Oxoid) plates as provided for double layer agar method. After the medium was allowed to solidify for 30 min at room temperature, the plates were incubated at 25°C and plaques were examined the next day.

#### Influence of temperature and UV light on bacteriophages activity

Bacteriophages were evaluated for their suppressive abilities and survival under two different abiotic stress conditions and tested for stability at a range of different temperatures (-80°C, −20°C, 25°C, 4°C, 37°C and 65°C) and under UV light at a wavelength of 375 nm (Gelman Hawksley Universal UV lamp; UV dose 50 mJ cm^2^, 20 cm from the light source). Accordingly, 4 ml of a high titre of each bacteriophage was exposed to UV radiation for 5 or 10 min or incubated for 24 h at different temperatures (-80°C, −20°C, 25°C, 4°C, 37°C and 65°C) following inoculation with 100 μl of *Pectobacterium* spp. cultures standardized at a cell concentration of *ca*. 10^8^ cfu ml^-1^. Results were compared with a positive control (the same amount of bacteria inoculated with free-phage titre). The results were measured as the ratio of change in bacterial concentration after spiking with bacteriophages across time using a Jenway 6300 (Jenway®) spectrophotometer. The absorbance at OD_600_ was measured at 3 h intervals for 49 h in triplicate and averaged.

### Molecular characterization of bacteriophages

#### DNA extraction, purification, tagmentation and sequencing

Prior to DNA extraction, the aliquot of bacteriophage lysate was filter–sterilized using a 10 ml syringe barrel fitted with a 0.22 μm filter Millex® GV filter unit (Merck) and maintained at 4°C prior to analysis. Bacteriophage particles were concentrated and purified using the method reported previously [27].

For DNA extraction, Qiamp DNeasy Blood and Tissue kit (Qiagen) was used following manufacturers’ instructions. DNA extracts were tested and concentrations adjusted to 0.2 ng μl^-1^ using a Quantus fluorometer and Quantifluor dsDNA kit (Promega) following the manufacturer’s instructions. Agencourt®AMPure® magnetic beads (Beckman Coulter) and Nextra®XT Library Preparation (Illumina) kits were used following the manufacturer’s instructions. For tagmentation, 5 μl of diluted bacteriophage DNA was treated using the Nextra®XT Library Preparation (Illumina) kit following the manufacturer’s instructions. Next-generation sequencing (NGS) was performed using the MiSeq^™^ sequencer (Illumina) with v2 2 x 250 sequencing reagents (Illumina) following the manufacturer’s instructions for denaturation of a 2 nM library.

#### Genomic analysis

The obtained fastq raw reads of bacteriophage genomes of the forward and reverse were assembled using *de novo* using Geneious Prime version 2019.1.3 (Biomatters Ltd.). Assembled sequences were compared using blastn tool [28] with bacteriophage sequences available in GenBank using Geneious Prime version 2019.1.3 (Biomatters Ltd.) mapped and open reading frames (ORFs) were predicted using SnapGene® (GSL Biotech). Further analysis of predicted ORFs was conducted with BLASTp (NCBI) [29] tool using SnapGene® (GSL Biotech). The obtained genomes were additionally annotated with Rapid Annotation using Subsystem Technology (RAST) version 2.0 with RASTtk pipeline [30] accessed via the http://rast.nmpdr.org/ website with the default setting options. Further *in silico* analysis were performed for the presence of transfer tRNA and mRNA genes with the use of tRNAscan-SE using RAST [30], genes encoding for toxins and mycotoxins using ResFinder 3.1, ToxFinder 1.0 [31, 32] and Virulence Finder 2.0 [33].

### Biocontrol of soft rot

#### Semi–*in planta* experiment on potato tubers using monophage

A potato half–tuber assay modified from the method previously reported [13] was used to evaluate the growth inhibition of six isolated bacteriophages against three isolates of *P*. *atrosepticum* (P16, C2557 and P1B) and one isolate of *P*. *carotovorum* subsp. *carotovorum* (SR22) (S1 Table) through co–inoculation on potato tubers. The bacteriophage concentration used was *ca*. 10^8^ pfu ml^-1^. For each isolate of selected *Pectobacterium* spp. the concentrations were adjusted to *ca*. 10^8^ cfu ml^-1^ in NB (approximately OD_600nm_ = 0.2). Potato tubers were cut in half using a sterile knife. Each half–tuber was inoculated with 100 μl of bacteria suspension (*ca*. 10^8^ cfu ml^-1^) by injection into the tuber using a pipette and sterile tips and left to absorb for 20–30 min. After absorption of bacteria, 100 μl of bacteriophage *ca*. 10^8^ pfu ml^-1^ was injected into the same spot on the tuber using a pipette. NB was inoculated as a negative control instead of the bacterial and phage suspensions, and a positive control of bacteria, only, was also included. Three potato half–tubers, obtained from three different potato tubers, were used per treatment and allocated randomly. The bactericidal effect of the bacteriophage suspension on the potato tissue was measured after incubation for 48 h at 25°C, under humid conditions (50 ml of sterile water added on tissue to each box), by calculating the ratio of the weight of each half–tuber taken before inoculation, and afterwards, following removal, by scraping off the rotting tissue. Each box contained 12 half–tubers. Tubers were treated with one of the four different bacterial isolates (P16, C2557, P1B and SR22) (S1 Table) and co-treated with six selected bacteriophage (3 half–tubers per isolate), treated only with one of the four different bacterial isolates (positive control; 12 half–tubers per isolate) or only NB (negative control; 12 half–tubers). Each box was replicated twice within the experiment, and the experiment was replicated twice over time. Results from the three experiments were averaged and the quantity of macerated tissue determinate as quantity of macerated tissue (%) = 100 –(mass of macerated tissue (g) of potato tuber after incubation x 100/ mass of tuber (g) after removal.

#### Phage cocktail

Prior to formulation of a phage cocktail for *in vitro* and field trial experiments, six bacteriophages lysates (*ca*. 10^8^ pfu ml^-1^) were assessed for virulence against SRP and stability through overlay, spotting, UV, temperature and semi-*in planta* assays. The bacteriophage cocktail consisting of six tested lysates (φMA1, φMA1A, φMA2, φMA5, φMA6 and φMA7) was mixed with a ratio of 1:1:1:1:1:1, with each phage lysate adjusted to be *ca*. 10^8^ pfu ml^-1^. The phage cocktail was stored at 4°C until use for up to 48 months.

#### Phage cocktail evaluation *in vitro*

*Pectobacterium* suspensions of four isolates (P16, C2557, P1B and SR22) (S1 Table) were prepared using the method for liquid application of bacteria and adjusted to *ca*. 10^8^ cfu ml^-1^. The volume of 100 μl of this suspension was added to 4 ml of the phage cocktail in NB (*ca*. 10^8^ pfu ml^-1^) in a sterile cuvette. The OD_600_ was measured for 24 h using a UV/VIS spectrophotometer (Jenway, 6300) to assess suppressive activity of bacterial growth. As a control, a bacterial culture was inoculated with the same volume (100 μl) in NB without bacteriophage.

#### Semi–*in planta* experiment on potato tubers using phage cocktail

A potato half-tuber assay was performed to evaluate the phage cocktail efficacy against a mix of *P*. *atrosepticum* and *P*. *carotovorum* subsp. *carotovorum* (P16, C2557, P1B and SR22) (S1 Table) co–inoculated on tubers. The phage cocktail concentration was adjusted to *ca*. 10^8^ pfu ml^-1^ in sterile water. The bacterial density was adjusted to *ca*. 10^8^ cfu ml^-1^ in sterile demineralized water to produce a mixed suspension of both *P*. *atrosepticum* and *P*. *carotovorum* subsp. *carotovorum*. Potato tubers obtained locally were sterilized and inoculated with both the bacterial suspension and the phage cocktail as described for the monophage semi–*in planta* experiment. The protective effect of the phage- based cocktail on the potato tissue was measured after incubation for 48 h at 28°C, in a humid box, by calculating the ratio of the average diameter of rotten potato tissue around the wells co–inoculated with bacteria and bacteriophage to the average diameter of rotten tissue around the wells inoculated with only the bacterial mixture. Each box contained two replicates each of three tubers, and each box was replicated twice. The entire experiment was repeated twice over time. Results from the experiments were averaged and the area of macerated tissue assessed as area of macerated tissue (%) = 100 –(area of macerated tissue (cm) after incubation x 100/ area of tuber (cm).

#### Phage cocktail used for treatments in field conditions

Phage cocktail for field trial application was formulated using 1 ml of the phage cocktail (six equal proportions of each bacteriophages: φMA1, φMA1A, φMA2, φMA5, φMA6 and φMA7) and 500 μl of liquid bacterial suspension OD_600nm_ = 0.2 (approximately *ca*. 10^8^ cfu ml^-1^) in 800 ml of NB and made up to 1 l with sterile water. Phage cocktail-bacteria mixture was incubated overnight at 25°C with agitation (200 rpm). After overnight incubation, phage cocktail was separated from bacterial debris by filtration using a Millipore Express TM® Plus 0.22μm filter (Fisher Scientific) and collected into a sterile filter receiver flask (Fisher Scientific) connected to a general purpose vacuum pump (KNF–Neuberger) and stored prior to use at 4°C.

#### Potato tubers

Four cultivars commonly cultivated in Northern Ireland of high grade potato seed mini–tubers (British Queens, Dunbar Standard, Maris Piper and Amora) originating from Northern Ireland were used in field trials in 2016–2018. Potatoes were stored at 5°C prior to use in the field trial.

For semi–*in planta* experiments, potato tubers (average diameter size 4 cm) of cultivars Dunbar Standard and/or British Queen, Maris Piper and Amora (purchased from local retailers), were washed to remove excess soil, surface-sterilized in 1–13% sodium hypochlorite for 10 min, rinsed three times in distilled water and dried with tissue paper before performing semi–*in planta* experiments using the monophage and cocktail formulation.

#### Treatments used in field trials

Two different treatments methods of potato mini-tubers in the years 2016–2018 were used in the field trials, including vacuum–infiltration and soil drench (spraying) to evaluate the effect of application of the phage cocktail on potatoes (Table 1).

**Table 1:** Summary of field experiments performed in years 2016–2018 on potato tubers in Northern Ireland to control soft rot/blackleg using phage cocktail.

Year	Location^a^	No. of plants per plot X no. of plots	Number of applications^b^	
			VI^c^^,^^e^	SD^d^^,^^f^
2016	Belfast, Co. Antrim	40 x 8	1	4
2017	Crossnacreevy, Co. Down	40 x 8	1	4
	Greenmount, Co. Antrim	20 x 4	1	4
2018	Crossnacreevy, Co. Down	20 x 4	1	4
	Loughgall, Co. Armagh	20 x 4	1	4

^a^Location in Northern Ireland, UK.

^b^Number of applications during one growing season.

^c^Phage cocktail treatment on artificial bacterial inoculum (*P*. *atrosepticum*: P16, C2557 and P1B, *P*. *carotovorum* subsp. *carotovorum*: SR22) *ca*. 10^8^ cfu ml ^-1^ applied on tubers before planting through vacuum–infiltration.

^d^Phage cocktail treatment on potato tubers exposed on naturally low inoculum from environmental sources or infected seeds (approximately *ca*. 10^2^ cfu ml^-1^).

^e^VI—phage cocktail applied before planting by vacuum–infiltration.

^f^SD—spraying of soil by phage cocktail with first spraying after planting following 4 weeks (1l/week).

The vacuum–infiltration method was used for pre–treatment of high grade mini–tubers with *Pectobacterium* spp. only (positive control), NB (negative control), *Pectobacterium* spp. and phage cocktail (vacuum–infiltration treatment). For vacuum–infiltration treatment, mini–tubers were vacuum–infiltrated using a sterile vacuum desiccator filled with 2 l of phage cocktail connected to a general purpose vacuum pump for 1 h. After that time, the vacuum pump was turned off and tubers left to soak for 30 min. Following this, potatoes were left to dry at room temperature for 15 min and incubated overnight at 25°C under humid conditions. After approximately 24 h, mini tubers were vacuum–infiltrated for 1 h in 2 l of *Pectobacterium* cocktail composed of four isolates (SR22, P1B, P16 and C2557) (S1 Table) of two species (*P*. *atrosepticum* and *P*. *carotovorum* subsp. *carotovorum*), after which the vacuum pump was turned off and the tubers left to soak for 30 min. As a negative control, mini tubers were vacuum–infiltrated for 1 h in 2 l of NB and for positive control, mini tubers were vacuum–infiltrated for 1 h in 2 l of *Pectobacterium* spp. suspension and allowed to soak for 30 min. Following this, mini–tubers were subsequently surface dried at room temperature for 15 min and incubated overnight at 25°C under humid conditions.

For soil drench in 2016–2018, untreated high–grade mini tubers were sprayed at an application rate of 1 l per plot (approximately 1–1.5 m^2^) with phage cocktail once per week starting from the first day of planting in the soil and continuing for the four following weeks. In 2018, an additional treatment was added (suppression experiment); mini–tubers were pre–treated with a *Pectobacterium* inoculum before planting (S1 Table) using the vacuum–infiltration method described above. Mini–tubers were then sprayed with phage cocktail starting on the day of planting and continuing for the following 4 weeks.

#### Evaluation of efficacy of the phage cocktail *in vivo*

In the years 2016–2018, the hypothesis tested was that potato plants treated with the phage cocktail would show less disease (symptoms, and isolation of pathogen) than positive infected plants. Assessment of the difference between the two different methods of application was also investigated assessed for emergence, soft rot/blackleg incidence and yield after harvest (mass and tubers number).

#### Persistence of phage cocktail treated tubers in field trial 2016 after harvest

To supplement knowledge about bacteriophage persistence and biology for extended periods of time, progeny from seed potatoes, treated using the phage cocktail through vacuum–infiltration and soil drench (spraying) of four cultivars (Amora, British Queen, Dunbar Standard and Maris Piper) planted in the first field trial in 2016 were further tested *in vitro* after harvest. The tubers were harvested after the growing season (May–October 2016) and stored at 5°C prior to the experiment performed between February and March 2017 using a whole tuber assay to assess the persistence and protective effect of the phage cocktail. Each whole tuber was inoculated with a bacterial suspension (except for the negative controls) using the method described for semi–*in planta* experiments above. As a negative control, untreated potatoes harvested from the field trial in 2016 were selected and inoculated with NB instead of bacterial suspension. Three potato tubers were used per treatment. The protective effect of the phage cocktail used in the field trial 2016 in progeny potatoes was determined after incubation for 48 h at 28°C in a humid box by measuring the amount of macerated tissue after incubation. Two replications were included in separate boxes in each experiment, and each experiment was repeated twice over time. Results from the experiments were averaged and the quantity of macerated tissue Qmt (mass) (%) was calculated using formula for mass in semi–*in planta* experiment on potato tubers using monophage.

### Statistical analysis

Statistical analysis was carried out using GenStat release 16.2 software (VSN, International) or NCSS 12 Data Analysis, LLC (Utah, USA). Analysis of variance one–way (ANOVA) was used to compare effectiveness of the treatments in terms of yield, emergence and soft rot/blackleg symptoms *in vivo* and mass/area of rotting tissue in bioassays. Multiple comparisons were performed using Fisher’s least significant difference (LSD).

## Results

### Isolation, purification and identification of bacteriophages

**Fig 1:**
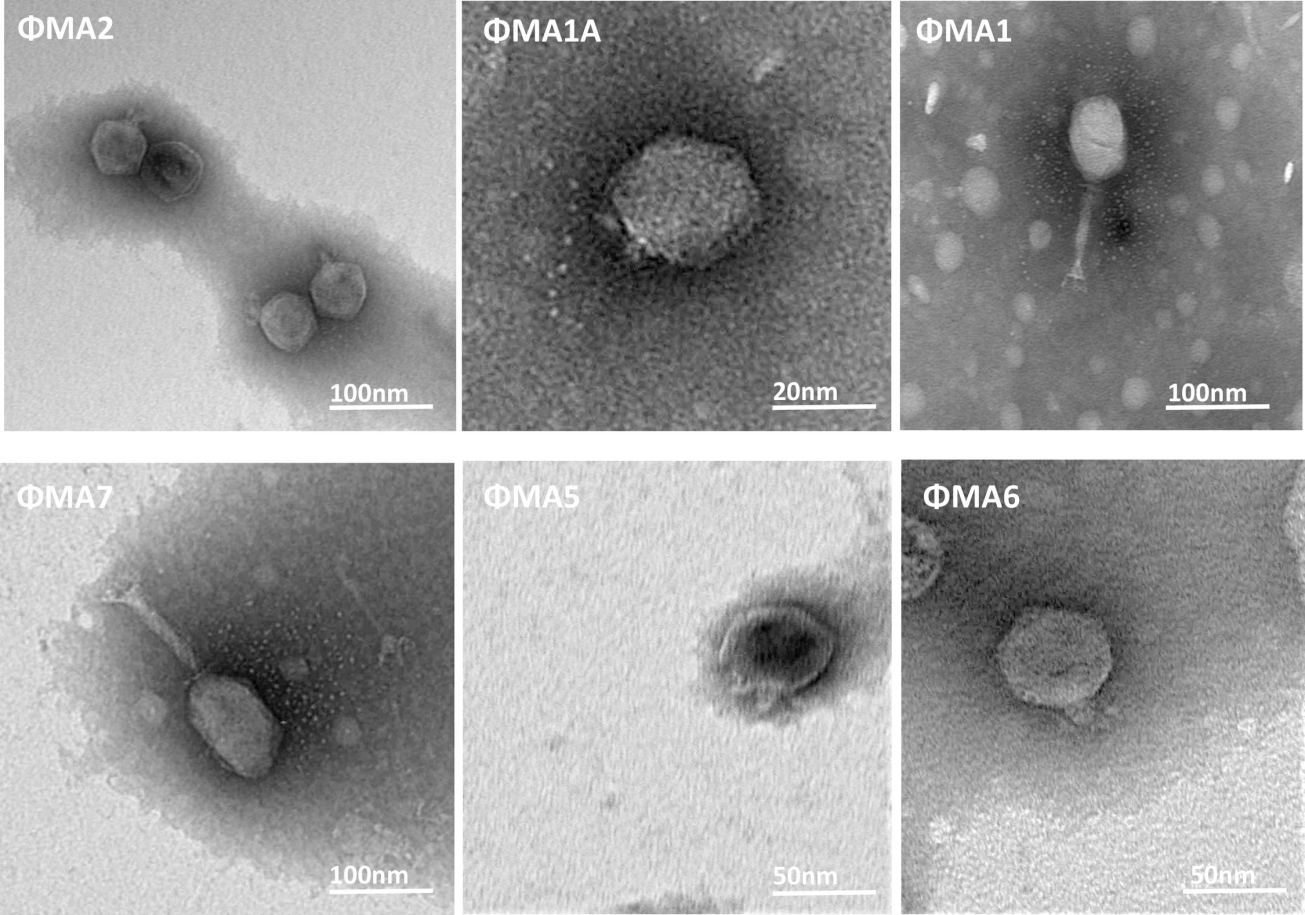
Transmission electron micrographs of six negatively stained (4% ammonium molybdate) bacteriophages isolated in this study belonging to two families of *Caudovirales* order. Bacteriophages of *Podoviridae* family: φMA2, φMA5, φMA1A, φMA6, and *Myoviridae* family bacteriophages: φMA7 and φMA1.

Six bacteriophages were recovered from processing water samples, purified and titrated. Examination using TEM (100 nm) revealed bacteriophages belonging to two families of order *Caudovirales*: *Podoviridae* and *Myoviridae* (Fig 1).

Four of the detected bacteriophages belonged to the *Podoviridae* family with heads of *c*. 50.2 nm x 51.3 nm–56.29 nm x 54.86 nm and short non-contractile tails up to 14.2 nm (Table 2). Two bacteriophages were characterized as members of the *Myoviridae* family with larger icosahedral heads (70.15 nm x 80.73 nm–61.70 nm x 113.6 nm) and rigid contractile tails (111.5 nm–113.9 nm) (Table 2).

**Table 2:** Characteristics of six isolated bacteriophages by visual assessment of plaque morphology and determination of morphotype using TEM.

No.	Phage	Plaque description^a^	Head diameter (nm)	Tail length (nm)	Order^b^	Family^b^
1.	φMA1	small, transparent	80.73 x 61.70	111.5	*Caudovirales*	*Myoviridae*
2.	φMA1A	medium, semi-transparent	55.34 x 53.31	short, non-contractile	*Caudovirales*	*Podoviridae*
3.	φMA2	medium, semi-transparent	56.29 x 54.86	short, non-contractile	*Caudovirales*	*Podoviridae*
4.	φMA5	big, semi-transparent	51.64 x 54.77	14.2	*Caudovirales*	*Podoviridae*
5.	φMA6	small, semi-transparent	50.20 x 51.30	short, non-contractile	*Caudovirales*	*Podoviridae*
6.	φMA7	small, transparent	70.15 x 113.6	113.9	*Caudovirales*	*Myoviridae*

^a^Plaque morphology assessed in overlay assay [13].

^b^Phages morphology determined using TEM.

### Host range

Results from the overlay and spotting assays against isolated SRP (S1 Table) showed that all isolated bacteriophages were able to lyse isolates of *Pectobacterium* spp. with the φMA2 showing the broadest host range (Table 3).

**Table 3:** Host range of six bacteriophages isolated in this study.

Bacteria		Bacteriophages					
Species	Isolate	φMA1	φMA2	φMA5	φMA1A	φMA6	φMA7
*P*. *atrosepticum*	P4A	+	+	+	+	+	+
	P4B	N	+	N	+	N	N
	P4C/16	+	N	N	+	N	N
	P2A	N	+	+	N	N	N
	P2B	N	N	N	N	N	N
	P3A/16	N	+	N	N	N	N
	P1B	+	+	+	+	N	N
	C2557	+	+	+	+	+	N
	PM/Z4/15	+	+	+	+	+	+
	PM/Z6/15	N	+	+	N	N	N
	P16	+	+	+	+	+	+
	P48	N	+	N	+	N	N
	P18B	N	+	N	N	N	N
	P13B	N	+	N	+	N	N
	P1B/14	N	+	+	+	N	+
*P*. *carotovorum* subsp. *carotovorum*	SR22	+	+	+	+	+	+
	C2558	N	+	+	N	N	+
*Dickeya* sp.	C2559	N	N	N	N	+	+

N—no lysis obtained in overlay and plaque assay. ‘+’—bacteria lysis.

### Influence of temperature and UV light on activity of bacteriophages and phage cocktail against *Pectobacterium* species

Differences in suppression of bacteria by bacteriophages were observed after 24 h incubation at -20°C, 25°C, 37°C and 65°C (S1A–S1G Fig). Bacteriophages φMA1, φMA2 and φMA5 incubated at -20°C, φMA1 and φMA5 incubated at 25°C and two bacteriophages incubated at higher temperatures (φMA2 at 65°C and φMA6 incubated at 37°C) suppressed bacterial growth (S1B and S1D–S1F Fig).

UV irradiation for 10 min inactivated only two bacteriophages (φMA2 and φMA1) up to 5 h after spiking with bacteria (S2D and S2B Fig), with suppression of bacteria growth by φMA1A, φMA5, φMA6 and φMA7 bacteriophages during 49 h of experiment (S2C and S2E–S2G Fig).

### NGS of bacteriophage’s genomes

Analysis of three bacteriophage genomes revealed that they belong to the order *Caudovirales* with the highest similarity of φMA1A, φMA6 to the *Autographivirinae* subfamily *Teseptimavirus* (*Pectobacterium* phage PP81) and φMA2 to *Phimunavirus* (*Pectobacterium* phage PP16) within the *Podoviridae* family (Table 4). The bacteriophage genomes were deposited in GenBank under the following accessions numbers MN271656, MN308080 and MN327636.

**Table 4:** Genome sizes and pairwise identities of bacteriophages determined using NGS.

Accession no.	Phage	Order	Family^b^	Subfamily^b^	Morphotype^b^	Id^a^ (%)	Reads no.	Genome (bp)
MN271656	φMA2	*Caudovirales*	*Podoviridae*	*Autographivirinae*	*Phimunavirus*	95.7	14,285	41,857
MN308080	φMA1A	*Caudovirales*	*Podoviridae*	*Autographivirinae*	*Teseptimavirus*	85.3	12,741	39,781
MN327636	φMA6	*Caudovirales*	*Podoviridae*	*Autographivirinae*	*Teseptimavirus*	85.5	17,592	38,553

^a^Percentage identity determinate using BLAST [28] as first hit available in GenBank.

^b^Family, subfamily and morphotype identified using BLAST [28].

### Genomic and *in silico* proteomic analysis

ORFs were identified for assembled genomes of *Podoviridae* bacteriophages to be between 51–55 with 80–90% identified to encode unique proteins with reliable identities (e–value > 0.001) to the available GenBank accessions entries. Annotated genomes revealed coding for hypothetical proteins mostly related to *Pectobacterium* phages PP16 and PP81, structural, replication and lifecycle proteins (S3–S5 Figs).

Several proteins used in DNA replication systems and suppression of the host were identified, including DNA polymerases and helicases (φMA1A, φMA2 and φMA6) (S3–S5 Figs), primases, suppression proteins (φMA1A, φMA2 and φMA6) (S3–S5 Figs) and endonucleases (φMA1A, φMA2 and φMA6) (S3–S5 Figs). Several genes that encode lysis proteins, indicating the lytic lifecycle of bacteriophages, were identified in bacteriophages φMA1A and φMA6, including those that encode lysin (φMA1A and φMA6), or lysozyme proteins (φMA2) (S3–S5 Figs). In addition, the characteristic type of endolysin (i.e. lysin N–acetylmuramoyl–L–alanine and holin class II) which is involved in the cell lysis process was identified in three of the bacteriophages genomes (S3–S5 Figs). Within the *Podoviridae* family, five proteins were identified to be involved in formation of the virions of isolated bacteriophage genomes. This included phage collar, capsid, tail fiber and phage internal proteins (S3–S5 Figs).

Annotated genomes did not revealed the presence of tRNAs/mRNA bacterial transcripts and genes that encode antibiotic resistance or toxins (S2 Table).

### Biocontrol of soft rot

#### Evaluation of phage cocktail *in vitro*

**Fig 2:**
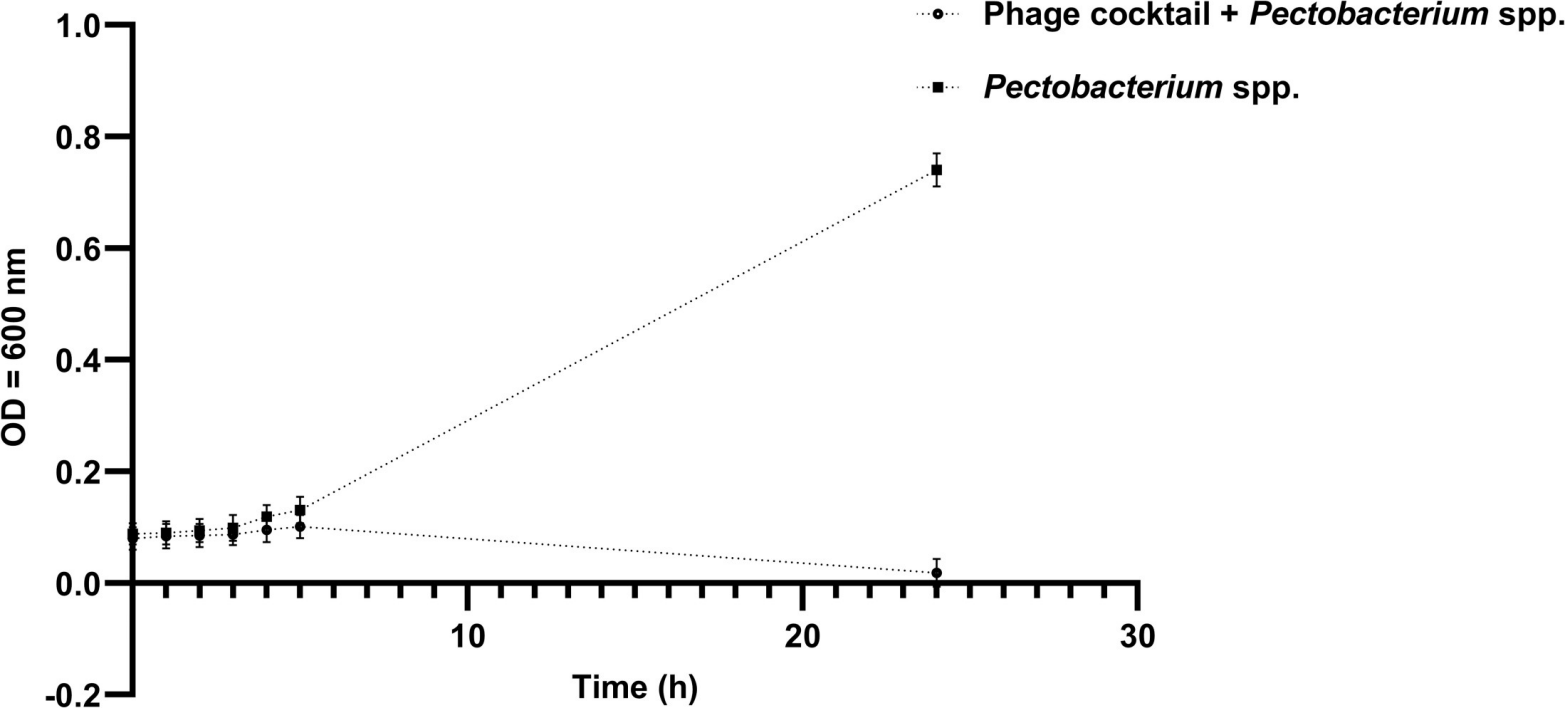
Suppression of *Pectobacterium* spp. by phage cocktail measured as change in absorbance at OD = 600 nm.

The formulated phage-based cocktail was efficacious to suppress bacterial growth in a 24 h experiment with significant inhibition of *Pectobacterium* growth after 4 h of the experiment (Fig 2).

#### Semi–*in planta* experiments on potato tubers using single bacteriophages and phage cocktail

**Fig 3:**
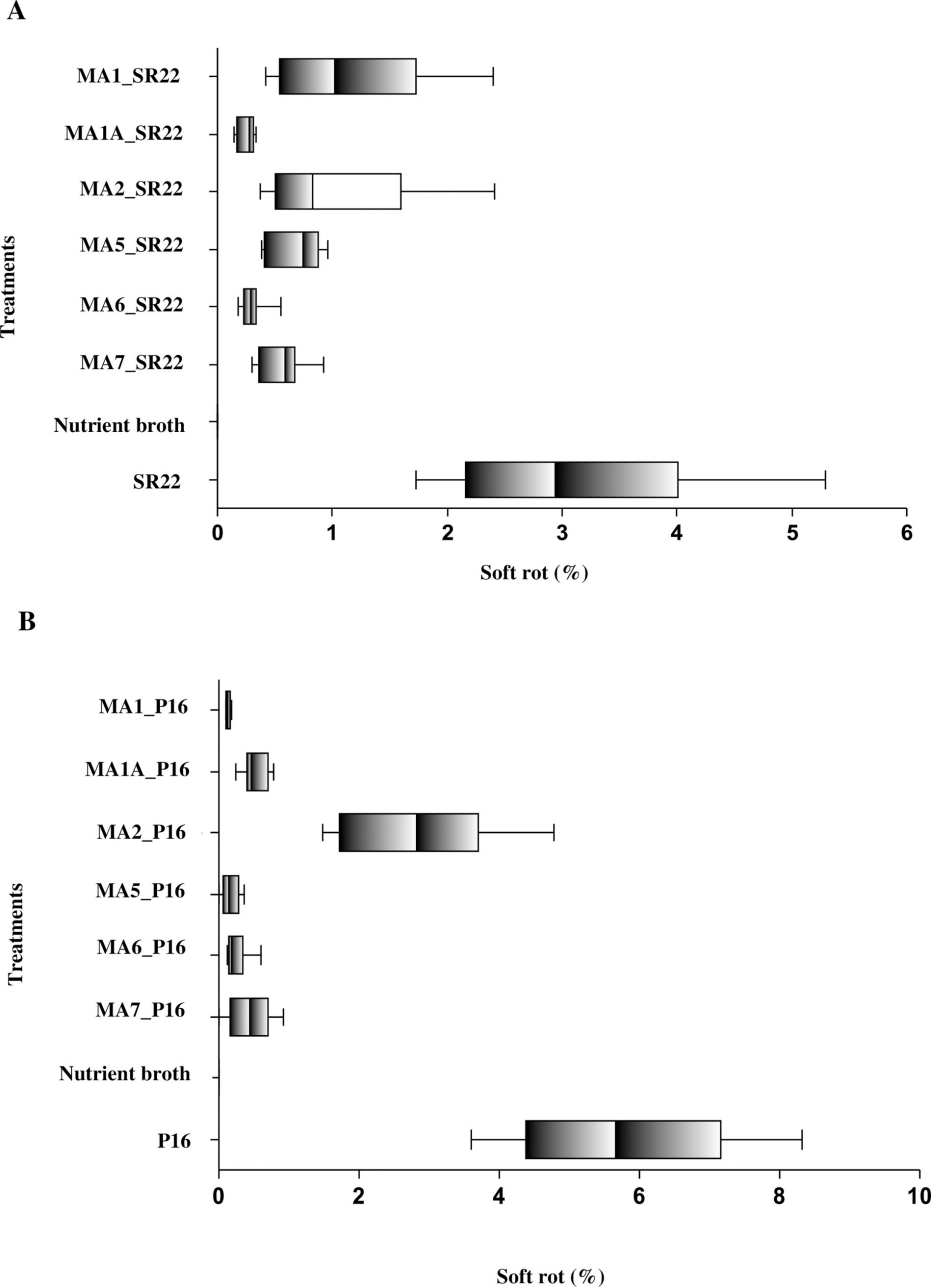
Percentage of soft rot mass in potato tubers Dunbar Standard inoculated in semi*–in planta* experiment with *Pectobacterium* species and six bacteriophages φMA1, φMA1A, φMA2, φMA5, φMA6 and φMA7 tested individually for 48 h at 25°C in humid conditions. (A) SR22 (*P*. *carotovorum* subsp. *carotovorum*), (B) P16 (*P*. *atrosepticum*). As a negative control (NC) NB was used. Box–plots are significantly different based on a Fisher’s LSD multiple comparison test (p = 0.05). Experiment performed in triplicate (n = 3) with (n = 2) replication and repeated over time (n = 2).

All six bacteriophages significantly reduced tissue maceration caused by *P*. *atrosepticum* (isolate P16) (*F* (7, 162) = 67.4, *p* < 0.001) and *P*. *carotovorum* subsp. *carotovorum* (isolate SR22) (*F* (7, 162) = 67.3, *p* < 0.001) (Fig 3A and 3B). Fisher’s LSD test indicated a significant difference at α = 0.05 for all six bacteriophages against *P*. *atrosepticum* (P16) in comparison to the positive control (Fig 3B). Fisher’s LSD test also indicated a significant difference at α = 0.05 for all six bacteriophages against *P*. *carotovorum* subsp. *carotovorum* (SR22) in comparison to the positive control (Fig 3A).

**Fig 4:**
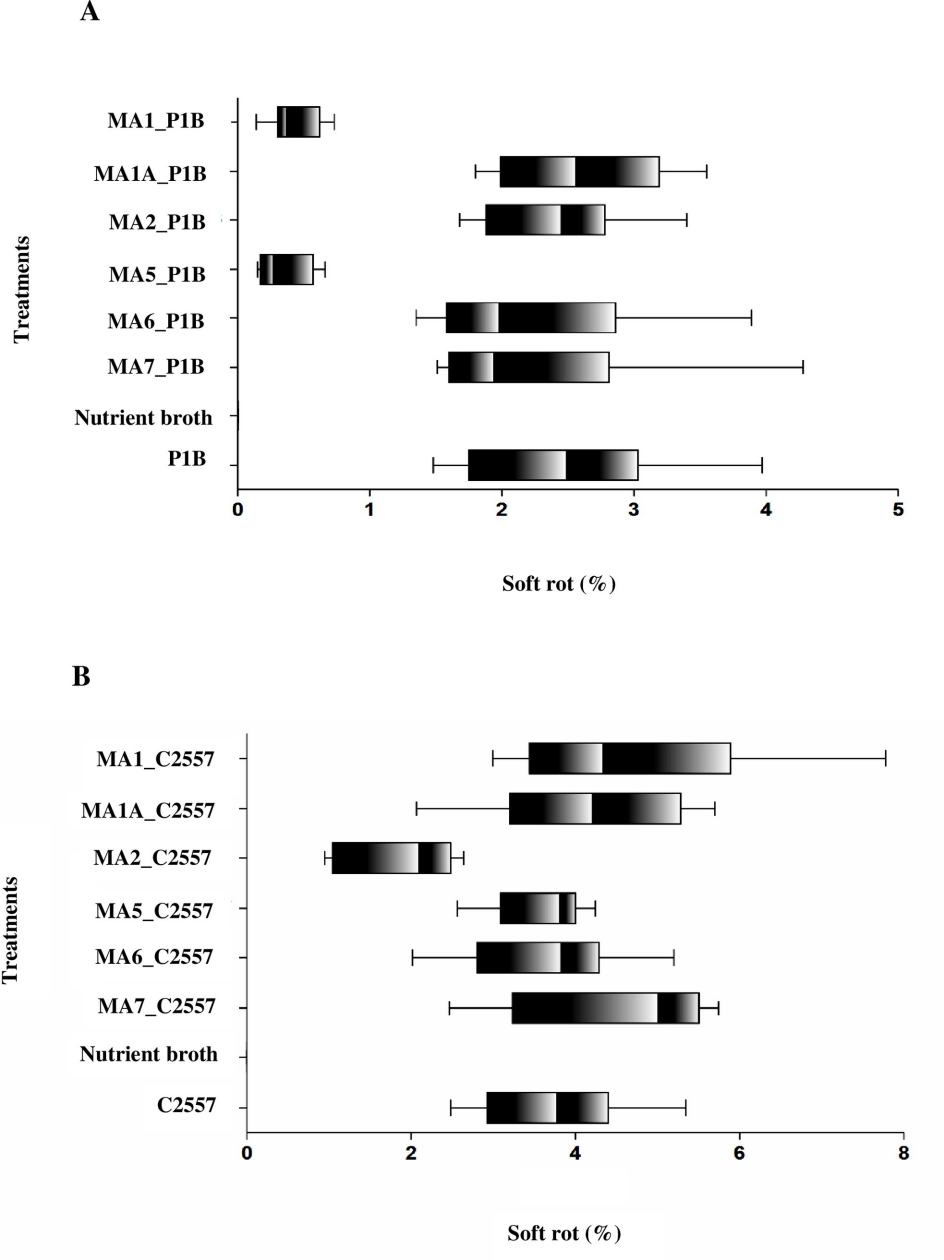
Percentage of soft rot mass in potato tubers Dunbar Standard inoculated in semi*–in planta* experiment with *Pectobacterium* species and six bacteriophages φMA1, φMA1A, φMA2, φMA5, φMA6 and φMA7 tested individually for 48 h at 25°C in humid conditions. (A) P1B *(P*. *atrosepticum)*, (B) C2557 (*P*. *atrosepticum*). As a negative control (NC) NB was used. Box–plots are significantly different based on a Fisher’s LSD multiple comparison test (p = 0.05). Experiment performed in triplicate (n = 3) with (n = 2) replication and repeated over time (n = 2).

Only φMA2 significantly supressed bacterial growth of the *P*. *atrosepticum* (C2557) isolate (*F* (7, 162) = 98.16), *p* < 0.001) (Fig 4B).

**Fig 5:**
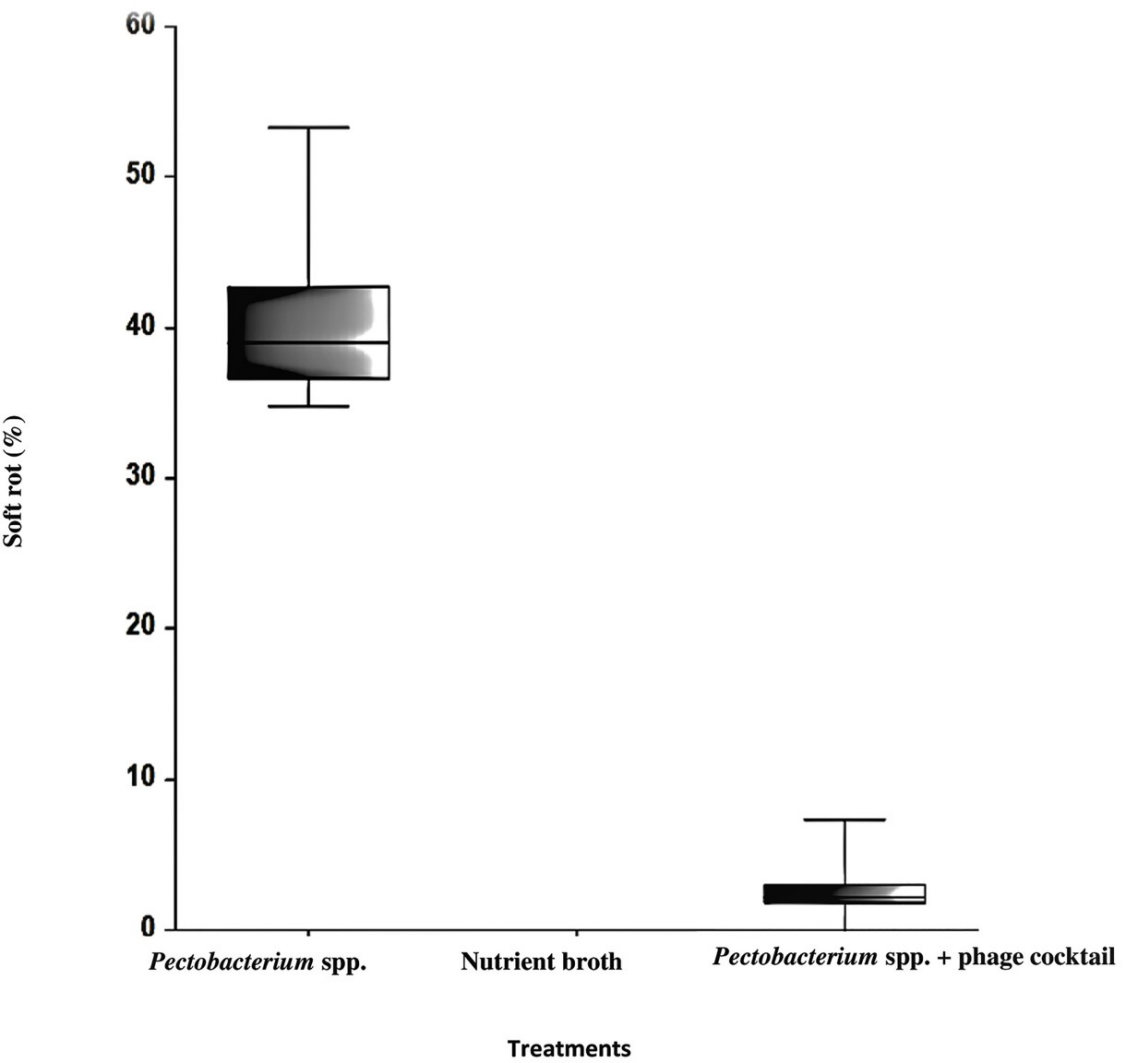
Percentage of soft rot area of potato tubers (four randomized cultivars including Dunbar Standard, British Queen, Amora and Maris Piper) in co-infection with phage cocktail and *Pectobacterium* spp. Only *Pectobacterium* spp. (*P*. *atrosepticum* and *P*. *carotovorum* subsp. *carotovorum*) as a positive control and sterile water as a negative control. Box–plots are significantly different based on a Fisher’s LSD multiple comparison test (p = 0.001). Experiment performed in triplicate (n = 3) with (n = 2) replication and repeated over time (n = 2).

Semi–*in planta* experiments on potato tubers using the phage cocktail revealed that six bacteriophages for the formulation of the phage cocktail significantly reduced the diameter of tissue maceration in the potato half tubers (2.6%) in comparison to positive controls (40.3%) (*P*. *atrosepticum* and *P*. *carotovorum* subsp. *carotovorum*) (Fig 5). There were statistically significant differences between the group means as determined by one-way ANOVA (*F* (2, 27) = 1297, *p* < 0.001). Fisher’s LSD multiple comparison test indicated that treatment with phage cocktail on tubers co-infected with *Pectobacterium* spp. had significantly less (α = 0.001) rotting than the positive control (Fig 5).

#### Evaluation of efficacy of the phage cocktail *in vivo*

In the field trial in 2016, there was a significant difference (*p* < 0.001) between the treatments in terms of emergence % (*F* (4, 40) = 11.2), mass of harvested tubers (*F* (4, 39) = 17.3), tuber number (*F* (4, 39) = 15.4) and soft rot % (*F* (4, 39) = 12.4) as determined by one-way-ANOVA. Fisher’s LSD test for multiple comparisons showed that the results from the treatments were significantly different (α = 0.05) (Table 5). Application of the phage cocktail through vacuum-infiltration did not increase the yield (emergence, mass or tuber number), and potatoes treated by this method were not significantly different in terms of percentage soft rot to the positive or negative control (Table 5). There was also no significant difference in yield (emergence, mass and tubers number) between the phage cocktail applied through soil–drench and untreated potatoes (Table 5). The negative control (NB) did not influence tuber yield, and no significant difference was observed between this treatment and phage cocktail treatment or the positive control applied by this method (Table 5). Those treated with phage cocktail showed significantly less soft rot assessed after harvest on tubers than untreated plants, the positive control and the negative control (Table 5). Blackleg was not visible in the plots during field assessment; however, a significant reduction of tuber emergence percentage was noted in all treatments, including the untreated potatoes with only 12 plants emerging from the 40 planted tubers.

**Table 5:** Means of potato tubers treatments—soil drench, vacuum-infiltration, negative control, untreated and positive control obtained from field trials 2016 (Belfast), 2017 (Crossnacreevy) and 2018 (Greenmount, Loughgall and Crossnacreevy) for emergence (%), yield: Mass of tubers after harvest (kg) and tuber number after harvest, soft rot after harvest /blackleg (%).

Treatment	Emergence^11^ (%)			Yield						Disease		
				Mass^7^ (kg)			Tuber no.^8^			Sr^9^/Bl^10^		
Means												
Year	2016	2017	2018	2016	2017	2018	2016	2017	2018	2016	2017	2018
Soil Drench^1^	28.4^	92.5^**	81.3	11.66**	32^**	4.9^**	130.5^	474^**	50.4**	0.6*/0	0/0	0.1**/0.4**
Soil Drench^2^	n/t	n/t	86.7^*	n/t	n/t	3.8**	n/t	n/t	52.9^**	n/t	n/t	0.4**/0.4**
Vacuum- Infiltration^3^	14	70.6**	73.3	5.4	22.5**	4.0**	60	365**	44.6**	0.9*/0	0.3*/0	2.9**/0.8**
Untreated	32	79.4	84.2	15.5	23.1	4.0	193.1	371	34.3	15/0	0.4/0	4.3/0.4
Negative Control^4^	18.8	52.2	74.6	7.0	16.0	3.9	77.9	290	36.3	0.9/0	0.2/0	3.0/0.8
Negative Control^5^	n/t	n/t	86.7	n/t	n/t	2.9	n/t	n/t	38.6	n/t	n/t	0.3/0
Positive Control^6^	18.1	43.1	73.3	2.0	11.0	2.2	16.6	178	29.0	0.4/0	0.6/0.1	23.1/10.4

^1^Untreated potato tubers sprayed with phage cocktail from planting up to 4 weeks (ones per week).

^2^Potato tubers inoculated with artificial *Pectobacterium* suspension *ca*.10^8^ cfu ml^-1^ using vacuum–infiltration and sprayed with phage cocktail from planting day up to 4 weeks (ones per week).

^3^Potato tubers inoculated with artificial *Pectobacterium* suspension *ca*.10^8^ cfu ml^-1^ and phage cocktail using vacuum–infiltration.

^4^Potato tubers inoculated with NB using vacuum–infiltration.

^5^Untreated potato tubers sprayed with NB from planting day up to 4 weeks (ones per week).

^6^Potato tubers inoculated with artificial *Pectobacterium* suspension *ca*.10^8^ cfu ml^-1^ using vacuum–infiltration.

^7^Mass of potato tubers harvested.

^8^Number of potato tubers harvested.

^9^Percentage of soft rot = number of plants with soft rot symptoms x 100% / total number of plants tested.

^10^Percentage of blackleg = number of plants with blackleg symptoms x 100% / total number of plants tested.

^11^Percentage emergence = number of plants assessed x 100% / number of plants planted. Asterisk (*) indicates a significant difference between the phage cocktail and positive control treatments according to Fisher’s least significant difference at

*P <* 0.05(*) and

*P* < 0.001(**).

Asterisk (^) indicates a significant difference between vacuum-infiltration and soil drench treatments according to Fisher’s least significant difference.

In the 2017 field trial, there was a significant difference (*p* < 0.001) in terms of emergence, weight, tuber number and blackleg incidence as determined by one-way-ANOVA between treatments and controls. A significant difference was indicated by the Fisher’s LSD test for multiple comparisons at α = 0.05 for potatoes treated with phage cocktail through soil drench. This treatment affected plant emergence, with 92% of planted tubers emerging, and a higher yield of harvested potatoes (mass and tuber counts) (Table 5). The treatment using vacuum-infiltration of the phage cocktail resulted in significantly higher yield assessed as emergence (number of plants), mass and number of tubers after harvest in comparison to the positive control (Table 5). In 2017, blackleg symptoms were visible in field trial plots in early July; phage treatments (vacuum-infiltration and soil drench) protected plants against disease development in the field and showed significantly reduced numbers of plants with symptoms in comparison to untreated plants and the positive control (Table 5). Assessment of the effects of four different cultivars (British Queen, Dunbar Standard, Amora and Maris Piper) indicated that there was no significant difference between the cultivars in terms of blackleg incidence.

The 2018 field trials, revealed significant effects of the treatments in terms of blackleg incidence (*F* (2, 66) = 2.30), tuber no. (*F* (2, 66) = 11.77), soft rot after harvest (*F* (2, 66) = 21.5) and mass of tubers (*F* (2, 66) = 19.07) determined by one-way-ANOVA at *p* < 0.001. Fisher’s LSD test indicated that response variables were significantly different at α = 0.05, in terms of the number of plants with blackleg and the number of tubers with soft rot symptoms for both vacuum-infiltration and soil drench treatments in comparison to the positive control. Both treatments showed a significantly higher yield in comparison to the positive control. There was no significant difference in emergence between all treatments and controls (*F* (2, 66) = 2.30, *p* < 0.04) (Table 5).

Application of NB through vacuum-infiltration, similar to 2017, significantly reduced yield (mass and number of tubers) compared with untreated potatoes (Table 5).

There was no effect of potato cultivar on any of the response variables measured, although British Queen (*p* < 0.001) (S3 Table) did produce higher tuber mass and numbers.

#### Persistence of phage cocktail treated tubers in field trial 2016 after harvest

**Fig 6:**
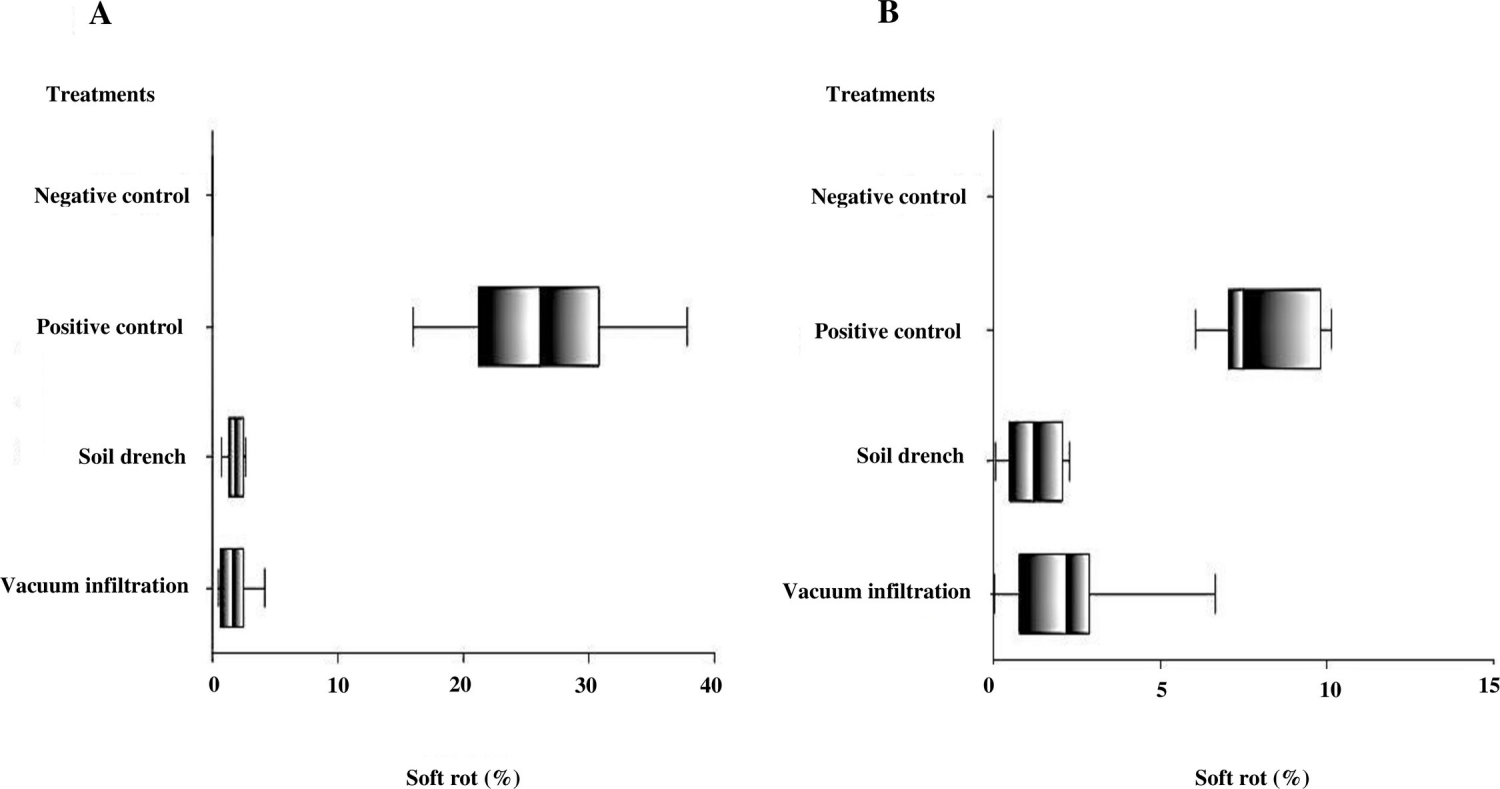
Percentage of soft rot on potato tubers (four randomized cultivars including: Dunbar Standard, British Queen, Maris Piper and Amora) after inoculation of mother tubers with a phage cocktail treatment from a field trial in 2016 and inoculated *in vitro* with *Pectobacterium* species. (A) Tubers inoculated with *P*. *carotovorum* subsp. *carotovorum* (SR22). (B) Tubers inoculated with mix of *P*. *atrosepticum* (P16, P1B and C2557). Only the *P*. *atrosepticum* mix or *P*. *carotovorum* subsp. *carotovorum* was inoculated as a positive control. NB inoculated as a negative control. Box–plots indicate significant differences based on Fisher’s LSD multiple comparison test (p = 0.05). Experiment performed in triplicate (n = 3) with (n = 2) replication and repeated over time (n = 2).

Progeny tubers from mother tubers treated with a phage cocktail through vacuum infiltration and soil drench in the field trial in 2016, and further inoculated with *P*. *atrosepticum* and *P*. *carotovorum* subsp. *carotovorum* showed significantly reduced amounts of macerated tissue (Fig 6A and 6B). There were statistically significant differences between the groups as determined by one-way ANOVA for *P*. *atrosepticum* (*F* (4, 36) = 50.9, *p* < 0.001) and for *P*. *carotovorum* subsp. *carotovorum* (*F* (4, 36) = 50.9, *p* < 0.001) (Fig 6A and 6B). Fisher’s LSD multiple comparison test indicated that all treatments with phage cocktail (vacuum–infiltration and soil drench) were significantly different at α = 0.05 in comparison to the positive control, providing suppression of both *P*. *atrosepticum* and *P*. *carotovorum* subsp. *carotovorum*.

## Discussion

Control of SRP in vegetables has been identified as a major challenge to food production and agricultural sustainability [34, 3]. In this study, laboratory and field tests indicated that bacteriophages from the families *Podoviridae* and *Myoviridae* were effective in controlling soft rot of potatoes caused by *P*. *atrosepticum* and *P*. *carotovorum* subsp. *carotovorum* as a phage cocktail formulation.

In this study, isolated bacteriophages infecting SRP belong to the *Podoviridae* and *Myoviridae* families. Likewise, several *Podoviridae* bacteriophages infecting *Pectobacterium* spp. have been described: ϕPP1 and ϕPPWS1 infecting *P*. *carotovorum* subsp. *carotovorum* [17, 35]; ϕPeat1, ϕM1, ϕCB1, ϕCB3 and ϕCB4 infecting *P*. *atrosepticum* [36, 37, 5]; and ϕA38 and ϕA41 infecting *P*. *parmentieri* [21]. These findings suggest that *Podoviridae* family bacteriophages are not as frequently isolated (only 14% from 6300 identified phages) [38] as bacteriophages from the family *Myoviridae*; however, they can be recovered against SRP more frequently.

This study tested potato processing water samples (probably directly related with infected tissue and soil) and therefore there was a higher chance that such samples contained potato–pathogen specific bacteriophages. However, this source has been indicated already as a rich reservoir for isolation of bacteriophages of *P*. *atrosepticum* and *P*. *parmentieri* [39, 40].

The stability of biocontrol products is an important factor underlying adoption in agriculture [10]. Several bacteriophages were exposed to high intensity (α = 375 nm) UV light with results overall indicating that the phages suppress SRP growth measured up to 49 h as a change in absorbance. Smolarska and co–workers [21] found that two *Podoviridae* family bacteriophages infecting *P*. *parmentieri* could not survive UV exposure for 5 and 10 min [21]. Czajkowski and co–workers [13] reported that *Myoviridae* family bacteriophages were unable to survive 10 min of UV radiation; however, 5 min of UV exposition reduced their concentration by about 50% as counted in plaque assay [13]. Similar to this, another report showed limited survival of *Dickeya* phages after 2 min of exposure to UV light assessed as plaque assay [19]. This study has shown temperature to have varied effects on individual bacteriophages, with a trend for bacteriophages to be more stable under lower temperature. This was also demonstrated by Czajkowski and co–workers [13] who showed that bacteriophages were more stable at -20°C than– 4°C, with optimal temperature from 4°C–37°C. Alič and colleagues [19] pointed out that for long term storage the most optimal temperature is 4°C, which is also in agreement with others [41].

The semi–*in planta* potato bioassays for single bacteriophages and phage cocktail showed that isolated bacteriophages suppressed the growth of separate bacterial isolates and a mix of *P*. *atrosepticum* and *P*. *carotovorum* subsp. *carotovorum* and significantly protected against soft rot development. However, host specificity occurred against some bacterial isolates measured in single bacteriophage assays. This is similar to the findings of Czajkowski and co–workers [23], who reported broad–host range bacteriophages of *D*. *solani* capable of significantly reducing maceration of tubers caused by SRP. Furthermore, another study reported that two selected bacteriophages of *P*. *parmentieri* showed high inhibitive abilities against *P*. *parmentieri* in semi–*in planta* experiments [21]. Similar to previous studies showing efficacy of using phage cocktail against selected SRP [23, 5], bioassays showed that the phage cocktail of all six bacteriophages was more effective than the monophage formulation, as none of the single bacteriophages tested could suppress all four different isolates of *Pectobacterium*. Similar to the results presented in this research, recent work on bacteriophages of *D*. *solani* showed significant inhibition of maceration on tubers using a cocktail of six bacteriophages selected based on genetic distinctions evaluated using high throughput sequencing [4, 39].

Phage cocktails have already been highlighted as promising tool and successfully evaluated to control other economically important plant pathogens, such as *Erwinia amylovora* [42], *Xanthomonas campestris* pv. *vesicatoria* [43], *Xanthomonas axonopodis* pv. *citri*, *Xanthomonas citrumelo* [44] and *Ralstonia pseudosolanacearum* [45].

In this study, the phage cocktail contained six bacteriophages which were assessed for virulence and stability *in vitro* prior to formulation of a cocktail. Based only on host range (Table 3), the number of phages in a cocktail could be reduced i.e up to four which were the most virulent in this test. Nevertheless, the rationale to add bacteriophages φMA6 and φMA7 was that only these two phages were able to lyse *Pectobacterium* isolate C2559 (Table 3). Phages φMA5 and φMA2 were the only two which could lyse *Pectobacterium* isolate PM/Z6/15 (Table 3). The stability (e.g. UV) was also different between the assessed phages, with phage φMA2 inactivated in the first 5 h of UV experiments, which might indicate potential stability problems of a cocktail when exposed to UV-light. Additionally, phage φMA5 is one of two phages (except φMA1) which significantly reduced soft rot caused by *Pectobacterium* isolate P1B. Adding phage φMA5 made the cocktail more robust and stable against a broad range of isolates. Interestingly, an inconsistency between host range tests (overlay and spot assays) and in semi–*in planta* experiments was observed in terms of virulence against *P*. *atrosepticum* isolates C2557 and P1B. Only phage φMA2 suppressed significantly isolate C2557 and phages φMA5 and φMA1 suppressed isolate P1B in semi-*in planta* experiments; however, more phages were virulent against these two bacteria isolates in overlay and spot assays. These findings agree with previous studies [46] that suggested performing semi *in–planta* experiments prior to cocktail formulation. This phenomenon might be related to growing resistance during extended time of experiment or more favourable disease development conditions during the time of experiment [47].

The bioassay on tubers treated by phage cocktail in the field trial and artificially inoculated with *Pectobacterium* has shown that bacteriophages can survive in tubers for long periods of time after treatment and partially protect against *Pectobacterium* spp. infection. Thus far, limited evidence exists regarding the persistence of bacteriophages in storage. Czajkowski and co–workers [12] investigated persistence on tubers and soil *in vitro*, in which bacteriophages were recovered from potato tubers and soil after 28 days from inoculation, showing that bacteriophages were more persistent on tubers and in soil compared with on leaves due to environmental factors such UV radiation [12]. Potential future work should examine the persistence of phage treatments in the progeny of treated tubers, as it is known that other viruses (i.e potato virus Y) can be translocated from mother to progeny tubers [48]. In the case of bacteriophages, transfer is probably more likely outside of the plant (e.g in soil/water). This suggests that bacteriophage treated tubers might be protected from SRP in subsequent years to their treatment. The evidence of long term survival of bacteriophages sprayed directly into the soil [45], and the results of this research, may indicate that the phages applied by soil drench survive in the soil and can then be transferred to the surface of the new tubers.

*In vivo* biocontrol in the field trials carried out in 2016–2018 on potato tubers revealed a suppressive effect of the formulated phage cocktail on soft rot/blackleg development, with a positive effect on yield of potato in 2 years (2017–2018). In the 2016 field trial, there was low emergence of untreated plants (32%) and a number of cases of soft rot after harvest (15%). This might indicate natural in–field infection of the tubers. As high grade mini–tubers were used for the trial, it is unlikely they were infected with SRP; however, Toth and co–workers [49] indicated that first generation tubers are not always free from bacterial infection.

In 2018, blackleg disease developed more slowly and no significant reduction of tubers in the soil was observed in comparison to previous years (2016 and 2017). Easily recognized symptoms started in the period of intensive rainfall from August–September, with significant suppression caused by bacteriophages on treated plants. Similar to this, it has been indicated that if there are unfavourable conditions for SRP growth, no disease may occur even when blackleg-causing bacteria are present [50]. Similarly, instead of reduced emergence, blackleg occurred when potato seed rotted prior to the establishment of a plant, and this is also an important manifestation of blackleg [51].

Generalized transduction (presence of bacterial transcripts in viral genomes) is considered as a threat for phage–therapy due to transmission of genes responsible for host resistance or those encoding for antibiotic or toxins, and therefore phages capable of transduction (lysogenic lifecycle phages) are not recommended for use in any phage formulations [52]. Similar to previous studies involving SRP phages [19, 5, 40], analysis of the three bacteriophage genomes did not indicate the presence of host resistance genes assessed as the presence of bacterial gene transcripts (encoding for tRNA or mRNA) or genes encoding for toxins, mycotoxins or human pathogens (i.e shiga-toxins). Moreover, similar to previously reported *Pectobacterium* phages [5, 19] analysis of three *Podoviridae* bacteriophages (φMA2, φMA1A and φMA6) genomes indicated no signatures related to lysogenic lifecycles of these phages and the presence of lysis, replication and host suppression modules.

## Conclusions

This work has provided results which indicate that bacteriophages have potential for use as MPCA against potato soft rot causing SRP. The phage cocktail tested here could potentially be efficacious in countries where prevalence of the species *P*. *atrosepticum* and *P*. *carotovorum* subsp. *carotovorum* is expected to cause tubers soft rot and/or blackleg. However, optimization of application conditions of phage cocktail using vacuum-infiltration would be worthwhile to develop as this method could have promise in the production of SRP free potato seeds.

## Supporting information

S1 Table Bacterial isolates used in this study isolated from macerated potato tissue from Northern Ireland. (DOCX)

S2 Table Features of annotated bacteriophages genomes in this study by RAST, Virulence Finder, ResFinder 3.1 and ToxFinder 1.0. (DOCX)

S3 Table Means of treatments of four potato cultivars: Amora (A), Dunbar Standard (D), British Queen (BQ) and Maris Piper (MP): Soil drench, vacuum-infiltration, untreated, negative control and positive control obtained from field trial 2018 (from 3 locations: Loughgall, Crossnacreevy and Greenmount) for emergence number; mass (kg); tubers number; soft rot number and blackleg. (DOCX)

S1 Fig Effect of temperature on activity of bacteriophages *in vitro* against *Pectobacterium* spp. (*P*. *atrosepticum*: P16, P1B and C2557 and *P*. *carotovorum* subsp. *carotovorum*: SR22) *ca*. 10^8^ CFU mL^-1^ after 24 h incubation under range of temperatures (-80°C, -20°C, 25°C, 37°C, 65°C) measured as changes in absorbance at OD_600_. (A) Phage cocktail, (B) φMA1, (C) φMA1A, (D) φMA2, (E) φMA5, (F) φMA6, (G) φMA7. Only *Pectobacterium* mix (Bacteria) used as positive control and phage/phage cocktail as a control sample (Control). Bars indicated ± standard error.
(PDF)

S2 Fig Effect of UV (λ = 375 nm) on activity of bacteriophages and phage cocktail against *Pectobacterium* spp. (*P*. *atrosepticum*: P16, P1B and C2557 and *P*. *carotovorum* subsp. *carotovorum*: SR22) *ca*. 10^8^ cfu ml^-1^after 5 and 10 min radiation measured as changes in absorbance at OD_600_. **(**A) Phage cocktail, (B) Phage φMA1; (C) Phage φMA1A; (D) Phage φMA2; (E) Phage φMA5; (F) Phage φMA6; (G) Phage φMA7. Only *Pectobacterium* mix (Bacteria) used as positive control and phage/ phage cocktail as a control sample (Control). Bars indicated ± standard error.
(PDF)

S3 Fig Structural and functional annotation map of φMA2 bacteriophage (41 857 bp) for 55 ORFs encoding proteins. Different colours indicate coding for the following proteins: hypothetical (blue), structural proteins (orange), proteins for phage replication and lifecycle (pink).
(TIF)

S4 Fig Structural and functional annotation map of φMA6 bacteriophage (38 553 bp) for 51 ORFs encoding proteins. Different colours indicate coding for the following proteins: hypothetical (blue), structural proteins (orange), proteins for phage replication and lifecycle (pink)
(TIF)

S5 Fig Structural and functional annotation map of φMA1A bacteriophage (39 781bp) for 52 ORFs encoding proteins. Different colours indicate coding for the following proteins: hypothetical (blue), structural proteins (orange), proteins for phage replication and lifecycle (pink).
(TIF)

## References

[1] Devaux A, Kromann P, Ortiz O. Potatoes for sustainable global food security. Potato Res. 2014; 57: 185–199.

[2] Charkowski AO. The changing face of bacterial soft- rot diseases. Annu Rev Phytopathol. 2018; 56: 269–288. 10.1146/annurev-phyto-080417-045906 29958075

[3] Hadizadeh I, Peivastegan B, Hannukkala A, van der Wolf JM, Nissinen R. Biological control of potato soft rot caused by *Dickeya solani* and the survival of bacterial antagonists under cold storage conditions. Plant Pathol. 2018; 68: 297–311.

[4] Carstens AB, Djurhuus AM, Kot W, Hestberg Hansen L. A novel six-phage cocktail reduces *Pectobacterium atrosepticum* soft rot infection in potato tubers under simulated storage conditions. FEMS Microbiol Lett. 2019; 366: pii: fnz101 10.1093/femsle/fnz101 31095303

[5] Buttimer C, Hendrix H, Lucid A, Neve H, Noben JP, Franz K, et al Novel N4—like bacteriophages of *Pectobacterium atrosepticum*. Pharmaceuticals. 2018; 11: 10.3390/ph11020045 29757952 PMC6027278

[6] Buttimer C, McAuliffe O, Ross RP, Hill C, O’Mahony J, Coffey A. Bacteriophages and bacterial plant diseases. Front. Microbiol. 2017; 8: 34 10.3389/fmicb.2017.00034 28163700 PMC5247434

[7] Harper DR. Criteria for selecting suitable infectious diseases for phage therapy. Viruses. 2018; 10: 177; 10.3390/v10040177. PMC5923471 29621149

[8] Kortright KE, Chan BK, Koff JL, Turner PE. Phage therapy: a renewed approach to combat antibiotic-resistant bacteria. Cell HostMicrobe. 2019; 13: 219–232. 10.1016/j.chom.2019.01.014 30763536

[9] Malik DJ, Sokolov IJ, Vinner GK, Mancuso F, Cinquerrui S, Vladisavljevic GT, et al Formulation, stabilization and encapsulation of bacteriophage for phage therapy. Adv ColloidInterface Sci. 2017; 249: 100–133. 10.1016/j.cis.2017.05.014 28688779

[10] Svircev A, Roach D, Castle A. Framing the Future with Bacteriophages in Agriculture. Viruses. 2018; 10: 218 10.3390/v10050218 29693561 PMC5977211

[11] Abedon ST, García P, Mullany P, Aminov R. Editorial: Phage Therapy: Past, Present and Future. FrontMicrobiol. 2017; 8: 10.3389/fmicb.2017.00981 28663740 PMC5471325

[12] Czajkowski R, Smolarska A, Ozymko Z. The viability of lytic bacteriophage ΦD5 in potato-associated environments and its effect on *Dickeya solani* in potato (*Solanum tuberosum* L.) plants. PLoS ONE. 2017; 12: e0183200 10.1371/journal.pone.0183200 28800363 PMC5553641

[13] Czajkowski R, Ozymko Z, Lojkowska E. Isolation and characterization of novel soilborne lytic bacteriophages infecting *Dickeya* spp. biovar 3 (‘*D*. *solani*’). Plant Pathol. 2014; 63: 758–772.

[14] Czajkowski R. Bacteriophages of soft rot Enterobacteriacea- a mini review. FEMS Microbiol Lett. 2016; 363: fnv230 10.1093/femsle/fnv230 Epub 2015 Nov 30. 26626879

[15] Eayre CG, Bartz JA, Concelmo DE. Bacteriophages of *Erwinia carotovora* and *Erwinia ananas* isolated from freshwater lakes. Plant Dis. 1995; 79: 801–804.

[16] Adriaenssens EM, Van Vaerenbergh J, Vandenheuvel D, Dunon V, Ceyssens PJ, De Proft M, et al T4-related bacteriophage LIMEstone isolates for the control of soft rot on potato caused by ‘*Dickeya solani*’. PLoS ONE. 2012; 7: e33227 10.1371/journal.pone.0033227 22413005 PMC3296691

[17] Lim JA, Jee S, Lee DH, Roh E, Jung K, Oh C, et al Biocontrol of *Pectobacterium carotovorum* subsp. *carotovorum* using bacteriophage PP1. J Microbiol Biotechnol. 2013; 23: 1147–1153. 10.4014/jmb.1304.04001 23727798

[18] Soleimani-Delfan A, Etemadifar Z, Emtiazi G, Bouzari M. Isolation of *Dickeya dadantii* strains from potato disease and biocontrol by their bacteriophages. Braz J Microbiol. 2015; 46: 791–797. 10.1590/S1517-838246320140498 26413062 PMC4568865

[19] Alič Š, Naglič T, Tušek-Žnidarič M, Ravnikar M, Peterka M, et al Newly isolated bacteriophages from the Podoviridae, Siphoviridae, and Myoviridae families have variable effects on putative novel Dickeya spp. Front Microbiol. 2017; 8: 10.3389/fmicb.2017.01870 29033917 PMC5626979

[20] Lee DH, Lim JA, Lee J, Roh E, Jung K, et al Characterization of genes required for the pathogenicity of *Pectobacterium carotovorum* subsp. *carotovorum* Pcc21 in Chinese cabbage. Microbiology. 2013; 159: 1487–1496. 10.1099/mic.0.067280-0 PMC3749726 23676432

[21] Smolarska A, Rabalski L, Narajczyk M, Czajkowski R. Isolation and phenotypic and morphological characterization of the first *Podoviridae* lytic bacteriophages ϕA38 and ϕA41 infecting *Pectobacterium parmentieri* (former *Pectobacterium wasabiae*). E JPlant Pathol. 2018; 150: 413–425.

[22] Day A, Ahn J, Fang X, Salmond GPC. Environmental bacteriophages of the emerging Enterobacterial phytopathogen, *Dickeya solani*, show genomic conservation and capacity for horizontal gene transfer between their bacterial hosts. Front Microbiol. 2017; 8: 1654 10.3389/fmicb.2017.01654 28912766 PMC5582154

[23] Czajkowski R, Ozymko Z, de Jager V, Siwinska J, Smolarska A, Ossowicki A, et al Genomic, Proteomic and Morphological Characterization of Two Novel Broad Host Lytic Bacteriophages ΦPD10.3 and ΦPD23.1 Infecting Pectinolytic *Pectobacterium* spp.and *Dickeya* spp. PLoS ONE. 2015; 10(3): e0119812 10.1371/journal.pone.0119812 25803051 PMC4372400

[24] Lee J, Kim S, Park TH. Diversity of bacteriophages infecting *Pectobacterium* from potato fields. J Plant Pathol. 2017; 99: 453–460.

[25] Zaczek—Moczydłowska MA, Fleming CC, Young GK, Campbell K, O Hanlon R. *Pectobacterium* and *Dickeya* species detected in vegetables in Northern Ireland. Eur J Plant Pathol. 2019; 154: 635–647.

[26] Fortier LC, Moineau S. Morphological and genetic diversity of temperate phages. Appl Environ Microbiol. 2007; 73: 7358–7366. 10.1128/AEM.00582-07 17890338 PMC2168219

[27] Devaney R, Trudgett J, Trudgett A, Meharg C, Smith V. A metagenomic comparison of endemic viruses from broiler chickens with runting-stunting syndrome and from normal birds. Avian Pathol. 2016; 45: 616–629. 10.1080/03079457.2016.1193123 27215546 PMC7113909

[28] Altschul SF, Gish W, Miller W, Myers EW, Lipman DJ. Basic local alignment search tool. J Mol Biol. 1990; 215: 403–410. 10.1016/S0022-2836(05)80360-2 2231712

[29] BLASTP http://blast.ncbi.nlm.nih.gov/Blast.cgi?PAGE=Proteins.

[30] Brettin T, Davis JJ, Disz T, Edwards RA, Gerdes S, Olsen GJ, et al RASTtk: a modular and extensible implementation of the RAST algorithm for building custom annotation pipelines and annotating batches of genomes. Sci Rep. 2015; 10: 5:8365. 10.1038/srep08365 25666585 PMC4322359

[31] ResFinder 3.1 https://cge.cbs.dtu.dk/services/ResFinder/

[32] ToxFinder 1.0 https://cge.cbs.dtu.dk/services/ToxFinder/

[33] Virulence Finder https://cge.cbs.dtu.dk/services/VirulenceFinder/

[34] Czajkowski R, Pérombelon MCM, van Veen JA, van der Wolf JM. Control of blackleg and tuber soft rot of potato caused by *Pectobacterium* and *Dickeya* species: a review. Plant Pathol. 2011; 60: 999–1013.

[35] Hirata H, Kashihara M, Horiike T, Suzuki T, Dohra H, et al Genome sequence of *Pectobacterium carotovorum* phage PPWS1 isolated from Japanese horseradish [*Eutrema japonicum* (Miq.) Koidz] showing soft-rot symptoms. Genome Announcement. 2016; 4: e01625–15 10.1128/genomeA.01625-15 27103734 PMC4841149

[36] Kalischuk M, Hachey J, Kawchuk L. Complete genome sequence of phytopathogenic *Pectobacterium atrosepticum* bacteriophage Peat1. Genome Announc. 2015; 3: e00760–15. 10.1128/genomeA.00760-15 26272557 PMC4536668

[37] Blower TR, Chai R, Przybilski R, Chindhy S, Fang X, Kidman SE, et al Evolution of *Pectobacterium* bacteriophage ΦM1 to escape two bifunctional type III toxin-antitoxin and abortive infection systems through mutations in a single viral gene. Appl Environ Microbiol. 2017; 83: e03229–e03216. 10.1128/AEM.03229-16 28159786 PMC5377504

[38] Ackermann HW, Prangishvili D. Prokaryote viruses studied by electron microscopy. Arch Virol. 2012; 157: 1843–1849. 10.1007/s00705-012-1383-y 22752841

[39] Carstens AB, Djurhuus AM, Kot W, Jacobs-Sera D, Hatfull GF, Hansen LH. Unlocking the potential of 46 new bacteriophages for biocontrol of *Dickeya solani*. Viruses. 2018; 10: 10.3390/v10110621 30423804 PMC6267328

[40] Kabanova A, Shneider M, Bugaeva E, Ha VTN, Miroshnikov K, Korzhenkov A, et al Genomic characteristics of vB_PpaP_PP74, a T7-like *Autographivirinae* bacteriophage infecting a potato pathogen of the newly proposed species *Pectobacterium parmentieri*. ArchVirol. 2018; 163: 1691–1694. 10.1007/s00705-018-3766-1 29423549

[41] Jones JB, Jackson LE, Balogh B, Obradovic A, Iriarte FB, Momol MT. Bacteriophages for Plant Disease Control. Annu Rev Phytopathol. 2007; 45: 245–262. 10.1146/annurev.phyto.45.062806.094411 17386003

[42] Gill JJ, Svircev AM, Smith R, Castle AJ. Bacteriophages of *Erwinia amylovora*. Appl Environ Microbiol. 2003; 69: 2133–2138. 10.1128/AEM.69.4.2133-2138.2003 12676693 PMC154828

[43] Balogh B. Characterization and Use of Bacteriophages Associated with Citrus Bacterial Pathogens for Disease Control. Ph.D. Dissertation, Unversity of Florida; Gainesville, FL 2006.

[44] Balogh B, Canteros BI, Stall RE, Jones JB. Control of citrus canker and citrus bacterial spot with bacteriophages. Plant Dis. 2008; 92: 1048–1052. 10.1094/PDIS-92-7-1048 30769518

[45] Fujiwara A, Fujisawa M, Hamasaki R, Kawasaki T, Fujie M, Yamada T. Biocontrol of *Ralstonia solanacearum* by Treatment with Lytic Bacteriophages. Appl Environ Microbiol. 2011; 77: 4155–4162. 10.1128/AEM.02847-10 21498752 PMC3131639

[46] Hyman P, Abedon ST. Bacteriophage host range and bacterial resistance. Adv. Appl. Microbiol. 2010; 70: 217–248. 10.1016/S0065-2164(10)70007-1 20359459

[47] Xie Y, Wahab L, Gill JJ. Development and validation of microtiter plate-based assay for determination of bacteriophage host range and virulence. Viruses. 2018; 10: 189. 10.3390/v10040189 PMC5923483 29649135

[48] Dupuis B. The movement of potato virus Y (PVY) in the vascular system of potato plants. E J Plant Pathol. 2017; 147: 365–373.

[49] Toth IK, Humphris S, Brierley J, Skelsey P, Saddler G, Cahill G, et al Routes of blackleg contamination of high grade potato seed stocks by *Pectobacterium* species. 2016; http://www.potatoes.ahdb.org.uk.

[50] Charkowski AO. Biology and control *Pectobacterium* in potato. American JPotato Res. 2015; 92: 223–229.

[51] De Boer SH, Rubio I. Blackleg of potato. APSnet, The Plant Health Instructor 2004; 10.1094/PHI-I-2004-0712-01

[52] Fillol-Salom A, Alsaadi A, Sousa JAM, Zhong L, Foster KR, Rocha EPC, et al Bacteriophages benefit from generalized transduction. PLoS Pathog. 2019; 15(7): e1007888 10.1371/journal.ppat.1007888 31276485 PMC6636781

