# Supplementary material for: Phage cocktail containing Podoviridae and Myoviridae bacteriophages inhibits the growth of Pectobacterium spp. under in vitro and in vivo conditions
Source: PLoS One. 2020 Apr 2;15(4):e0230842. doi: 10.1371/journal.pone.0230842 (PMC7117878; doi:10.1371/journal.pone.0230842)
Supplement: S1 Table — (DOCX) [file pone.0230842.s001.docx]

| **No.** | **Isolate*** | **Location** | **Isolation source** | **Year** | **Identity** |
| --- | --- | --- | --- | --- | --- |
| 1. | P4A | Co. Antrim, NI | potato tuber | 2016 | *P. atrosepticum* |
| 2. | P4B | Co. Antrim, NI | potato tuber | 2016 | *P. atrosepticum* |
| 3. | P4C/16 | Co. Antrim, NI | potato tuber | 2016 | *P. atrosepticum* |
| 4. | P2A | Co. Antrim, NI | potato tuber | 2016 | *P. atrosepticum* |
| 5. | P2B | Co. Antrim, NI | potato tuber | 2016 | *P. atrosepticum* |
| 6. | P3A/16 | Co. Antrim, NI | potato tuber | 2016 | *P. atrosepticum* |
| 7. | P1B | Co. Antrim, NI | potato tuber | 2016 | *P. atrosepticum* |
| 8. | C2557 | Co. Antrim, NI | potato tuber | 2015 | *P. atrosepticum* |
| 9. | PM/Z-4/15 | Co. Antrim, NI | potato tuber | 2015 | *P. atrosepticum* |
| 10. | PM/Z-6/15 | Co. Antrim, NI | potato tuber | 2015 | *P. atrosepticum* |
| 11. | P16 | unknown, NI | potato tuber | 2014 | *P. atrosepticum* |
| 12. | P48 | unknown, UK | potato stem | 2014 | *P. atrosepticum* |
| 13. | P18B | unknown, UK | potato stem | 2014 | *P. atrosepticum* |
| 14. | P13B | unknown, UK | potato stem | 2014 | *P. atrosepticum* |
| 15. | P1B/14 | unknown, UK | potato stem | 2014 | *P. atrosepticum* |
| 16. | SR22 | unknown, NI | potato tuber | 2014 | *P. c.* subsp. *carotovorum* |
| 17. | C2558 | Co. Antrim, NI | potato tuber | 2015 | *P. c.* subsp. *carotovorum* |
| 18. | C2559 | Co. Antrim, NI | potato tuber | 2015 | *Dickeya* spp. |

*-Isolates from Agri-Food and Biosciences Institute collection, Belfast, Northern Ireland (NI), UK; identified as SRP using crystal violet pectate media, real-time/conventional PCR and/or *recA* gene sequences with the methods reported previously [47]. Following SRP isolates has been deposited in GenBank under accession numbers: P4A (MH290883), P4B (MH346385), P2A (MH481716), P2B (MH481717), P1B (MH329878), C2557 (MK673745), P16 (MH481722), SR22 (MK305814) and C2558 (MK305815).
