# Supplementary material for: Phage cocktail containing Podoviridae and Myoviridae bacteriophages inhibits the growth of Pectobacterium spp. under in vitro and in vivo conditions
Source: PLoS One. 2020 Apr 2;15(4):e0230842. doi: 10.1371/journal.pone.0230842 (PMC7117878; doi:10.1371/journal.pone.0230842)
Supplement: S2 Table — (DOCX) [file pone.0230842.s002.docx]

| **Bacteriophage** | | | |
| --- | --- | --- | --- |
|  |  |  |  |
| **Feature** | **φMA1A** | **φMA2** | **φMA6** |
|  |  |  |  |
| GC content (%)^a^ | 48.9 | 51.6 | 48.9 |
| L50^a^ | 1 | 1 | 1 |
| Number of contigs (with PEGs)^a^ | 1 | 1 | 1 |
| Number of subsystems (genes)^a^ | 5 | 5 | 5 |
| Number of coding sequences (ORFs)^a^ | 52 | 55 | 51 |
| Number of RNAs^a^ | 0 | 0 | 0 |
| Shiga-toxins^b^ | 0 | 0 | 0 |
| Exoenzymes/toxins^c^ | 0 | 0 | 0 |
| Virulence genes^d^ | 0 | 0 | 0 |
| Antibiotic resistance genes^e^ | 0 | 0 | 0 |
| Genes involved in mycotoxins synthesis^f^ | 0 | 0 | 0 |

^a^Features determinate by RAST.

^b^Features determinate by Virulence Finder 2.0.for identifying genes encoding for shiga-toxins produced by *Escherichia coli*.

^c^Features determinate by Virulence Finder 2.0 for identifying genes encoding for exoenzymes/toxins

produced by *Staphylococcus aureus*.

^d^Features determinate by Virulence Finder 2.0 for identifying virulence genes of *Listeria*, *S. aureus*, *E. coli, Enterococcus*.

^e^Features determinate by ResFinder 3.2 at threshold 90% for identifying acquired antimicrobial resistance genes for: aminoglycoside, beta-lactam, colistin, fluoroquinolone, fosfomycin, glycopeptide, MLS – macrolide, lincosamide, streptogramin B, nitroimidazole, oxazolidione, phenicol, rifampicin, sulphonamide, tetracycline and trimethoprim.

^f^Features determined by ToxFinder with default setting options for detection genes involve in synthesis of mycotoxins such as: aflatoxins, citrinins, patulins, ergots, fumonisins, ochratoxins and trichocethene.
