# Supplementary material for: Phage cocktail containing Podoviridae and Myoviridae bacteriophages inhibits the growth of Pectobacterium spp. under in vitro and in vivo conditions
Source: PLoS One. 2020 Apr 2;15(4):e0230842. doi: 10.1371/journal.pone.0230842 (PMC7117878; doi:10.1371/journal.pone.0230842)
Supplement: S3 Table — (DOCX) [file pone.0230842.s003.docx]

| **Treatment** | **Emergence number** | | | | **Mass (kg)** | | | | **Tubers number** | | | | **Blackleg (%)** | | | | **Soft rot (%)** | | | |
| --- | --- | --- | --- | --- | --- | --- | --- | --- | --- | --- | --- | --- | --- | --- | --- | --- | --- | --- | --- | --- |
|  |  |  |  |  |  |  |  |  |  |  |  |  |  |  |  |  |  |  |  |  |
|  |  |  |  |  |  |  |  |  |  |  |  |  |  |  |  |  |  |  |  |  |
|  | **A^1^** | **D^2^** | **MP^3^** | **BQ^4^** | **A^1^** | **D^2^** | **MP^3^** | **BQ^4^** | **A^1^** | **D^2^** | **MP^3^** | **BQ^4^** | **A^1^** | **D^2^** | **MP^3^** | **BQ^4^** | **A^1^** | **D^2^** | **MP^3^** | **BQ^4^** |
| Soil drench^5^ | 4.5 | 4 | 4.2 | 4.5 | 5 | 3.7 | 5.1 | 5.8 | 53.3 | 41.9 | 53 | 63.8 | 0.1 | 0 | 0 | 0 | 0.3 | 0 | 0 | 0 |
| Soil drench^6^ | 4.2 | 3.9 | 4.2 | 3.8 | 4 | 3.6 | 3.6 | 3.8 | 49.2 | 49.8 | 53 | 49.8 | 0 | 0 | 0 | 0.1 | 0 | 0.1 | 0 | 0 |
| Vacuum-infiltration^7^ | 3.5 | 3.8 | 3.5 | 3.6 | 4 | 3.3 | 3.9 | 4.9 | 47 | 38.2 | 40 | 53.4 | 0.2 | 0 | 0 | 0 | 0.2 | 0.1 | 0 | 0 |
| Untreated^8^ | 4.3 | 3.8 | 4.2 | 4.4 | 4 | 3.6 | 4 | 4.2 | 32.5 | 31.4 | 34 | 39 | 0 | 0 | 0 | 0.1 | 0.6 | 0.8 | 1 | 1 |
| Negative control^9^ | 4.2 | 4 | 4.6 | 4.3 | 4 | 3.6 | 4.2 | 4.4 | 40 | 34 | 40 | 40.7 | 0 | 0 | 0 | 0 | 0.2 | 0.1 | 0 | 0 |
| Negative control^10^ | 3.7 | 3.6 | 3.7 | 3.7 | 3 | 2.7 | 3 | 2.7 | 37.7 | 34.9 | 36 | 37 | 0.2 | 0 | 0 | 0 | 0.3 | 0.3 | 0 | 0 |
| Positive control^11^ | 4 | 3.5 | 3.6 | 3.4 | 2 | 2.3 | 2.4 | 2.2 | 27.5 | 24.9 | 29 | 34.5 | 0.8 | 0.3 | 0.3 | 0.8 | 4.3 | 3.4 | 4 | 5 |

^1^The potato variety–Amora (A).

^2^The potato variety–Dunbar Standard (D).

^3^The potato variety–Maris Piper (MP).

^4^The potato variety–British Queen (BQ).

^5^Soil drench–pre-treated potatoes inoculated with *P. atrosepticum* (P16, C2557 and P1B) and *P. carotovorum* subsp. *carotovorum* (SR22) and sprayed with phage cocktail.

^6^Soil drench–uninfected potatoes sprayed with phage cocktail.

^7^Pre–treated potatoes inoculated with *P. atrosepticum* (P16, C2557 and P1B) and *P. carotovorum* subsp. *carotovorum* (SR22) and treated with phage cocktail through vacuum–infiltration method.

^8^Untreated potato tubers.

^9^Potato tubers sprayed with NB.

^10^Pre–treated potato tubers with NB through vacuum–infiltration before planting.

^11^Pre–treated potato tubers with *P. atrosepticum* (P16, C2557 and P1B) and *P. carotovorum* subsp. *carotovorum* (SR22) through vacuum-infiltration before planting.
