# Supplementary figures and images for: Phage cocktail containing Podoviridae and Myoviridae bacteriophages inhibits the growth of Pectobacterium spp. under in vitro and in vivo conditions
Source: PLoS One. 2020 Apr 2;15(4):e0230842. doi: 10.1371/journal.pone.0230842 (PMC7117878; doi:10.1371/journal.pone.0230842)

**A**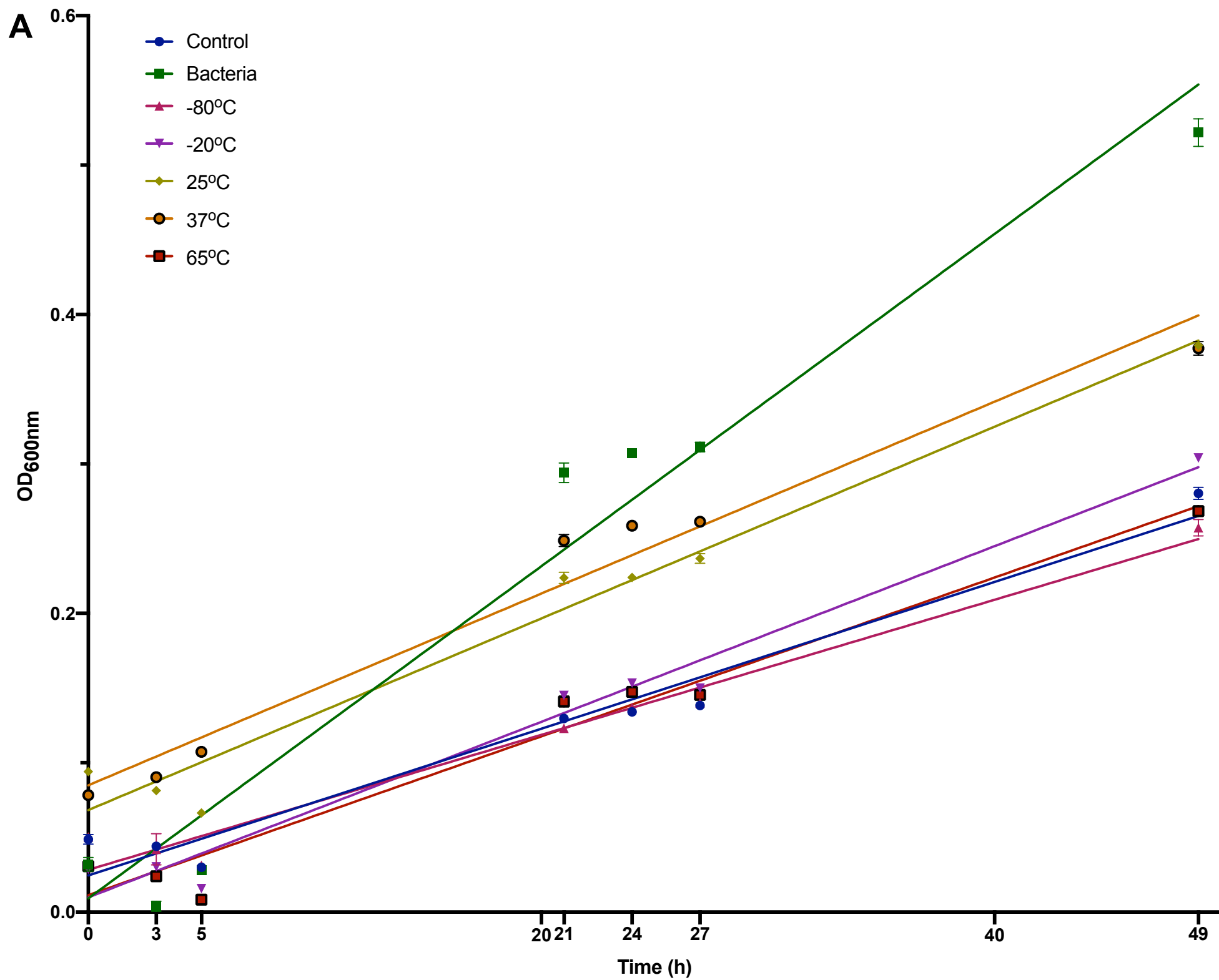

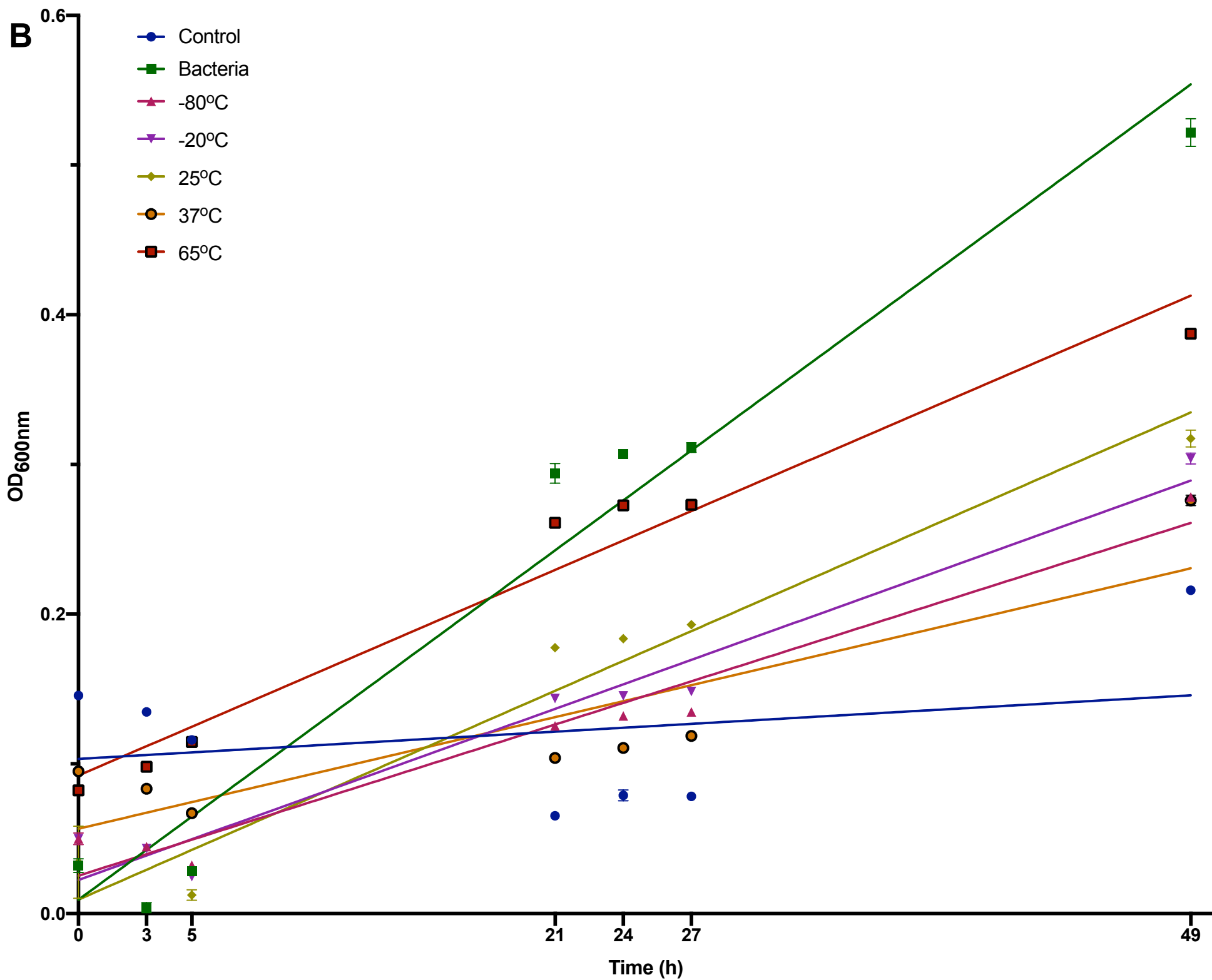

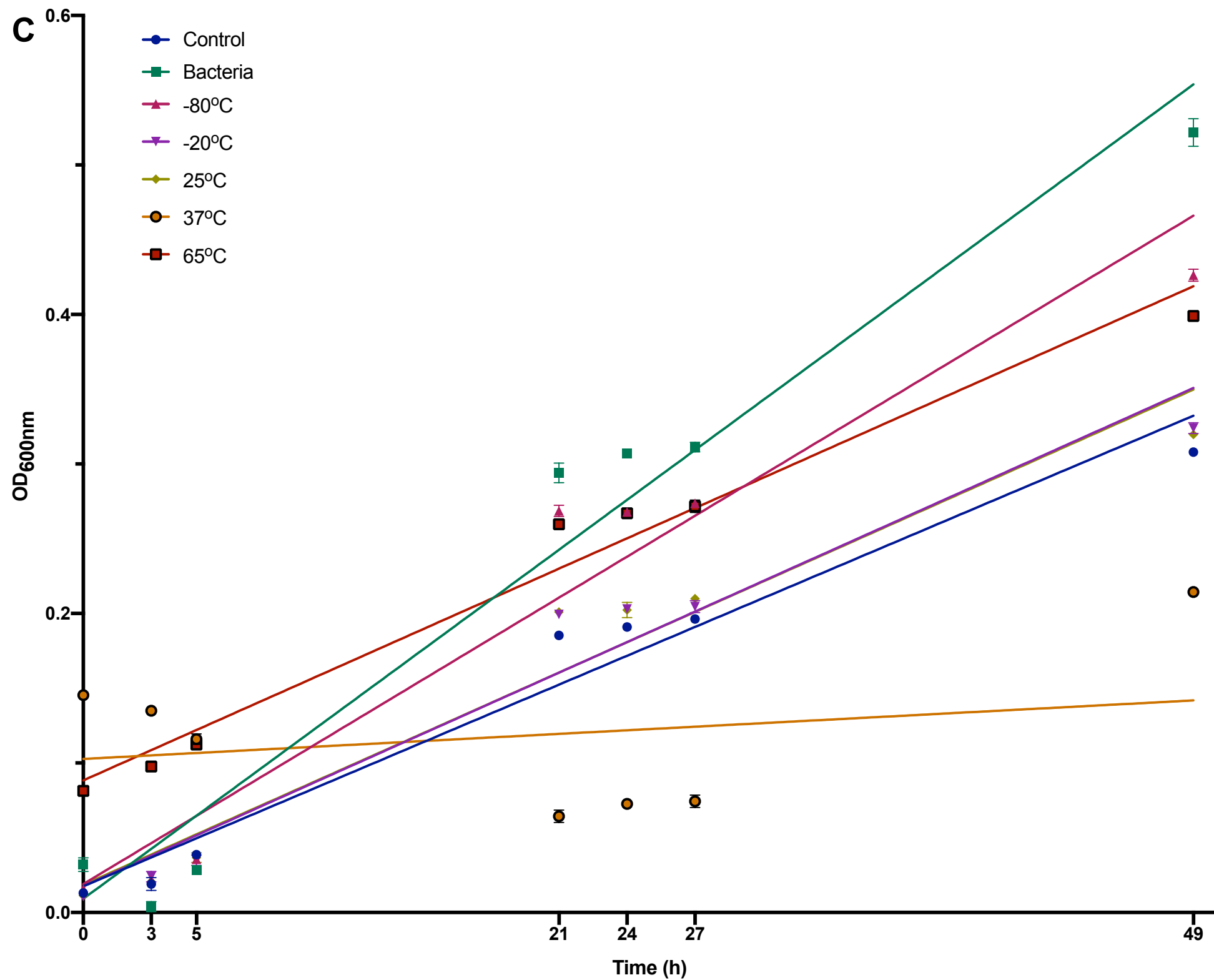

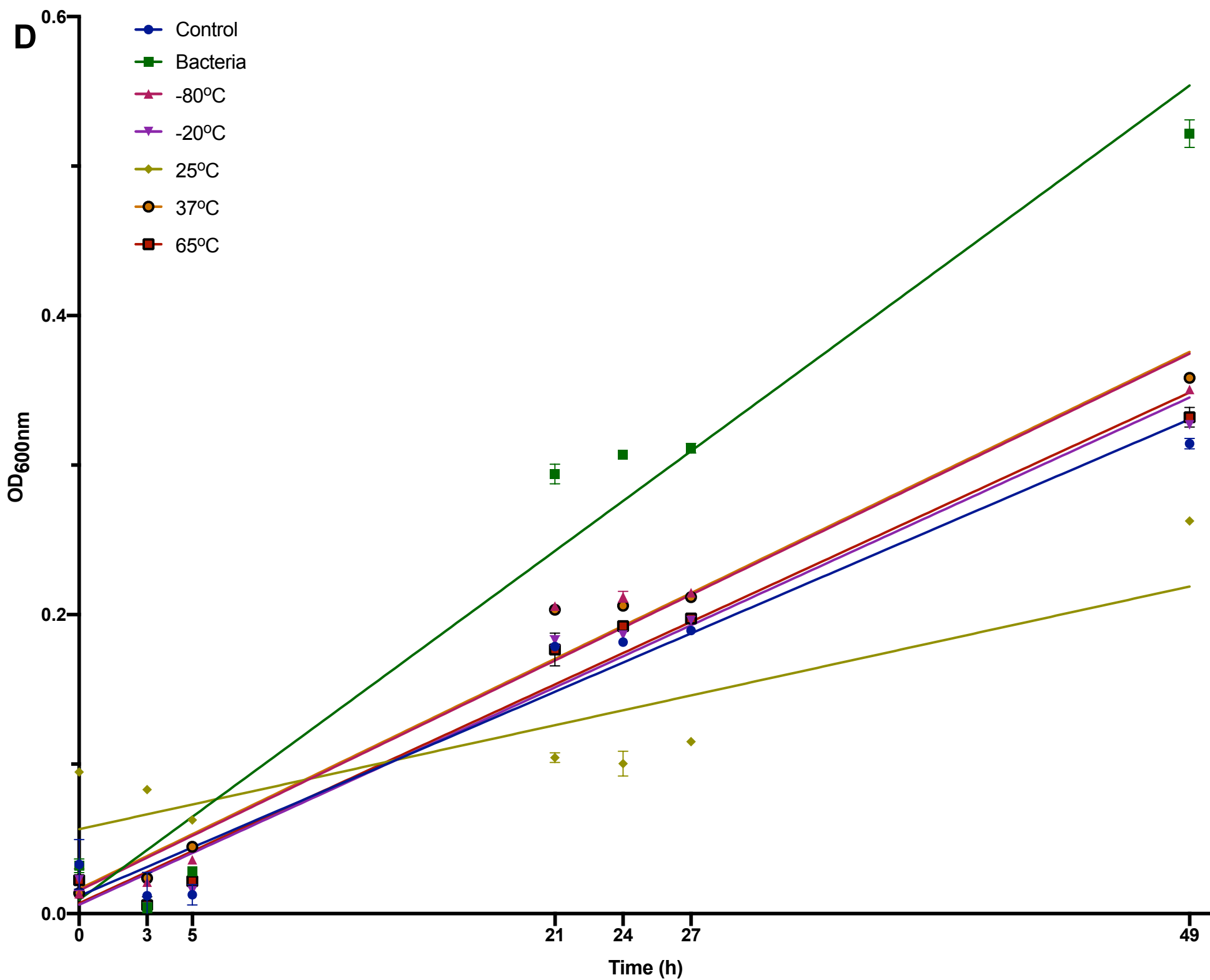

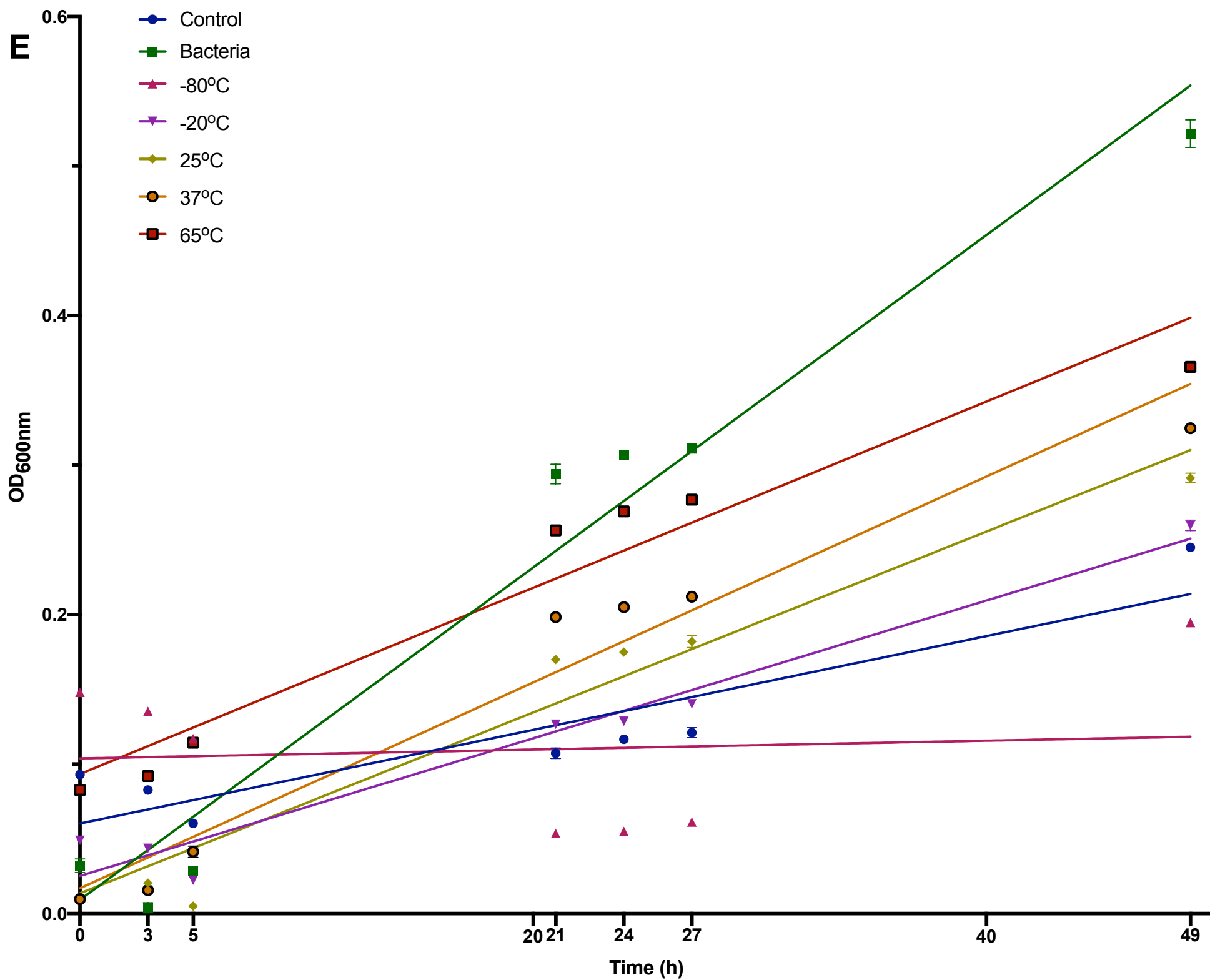

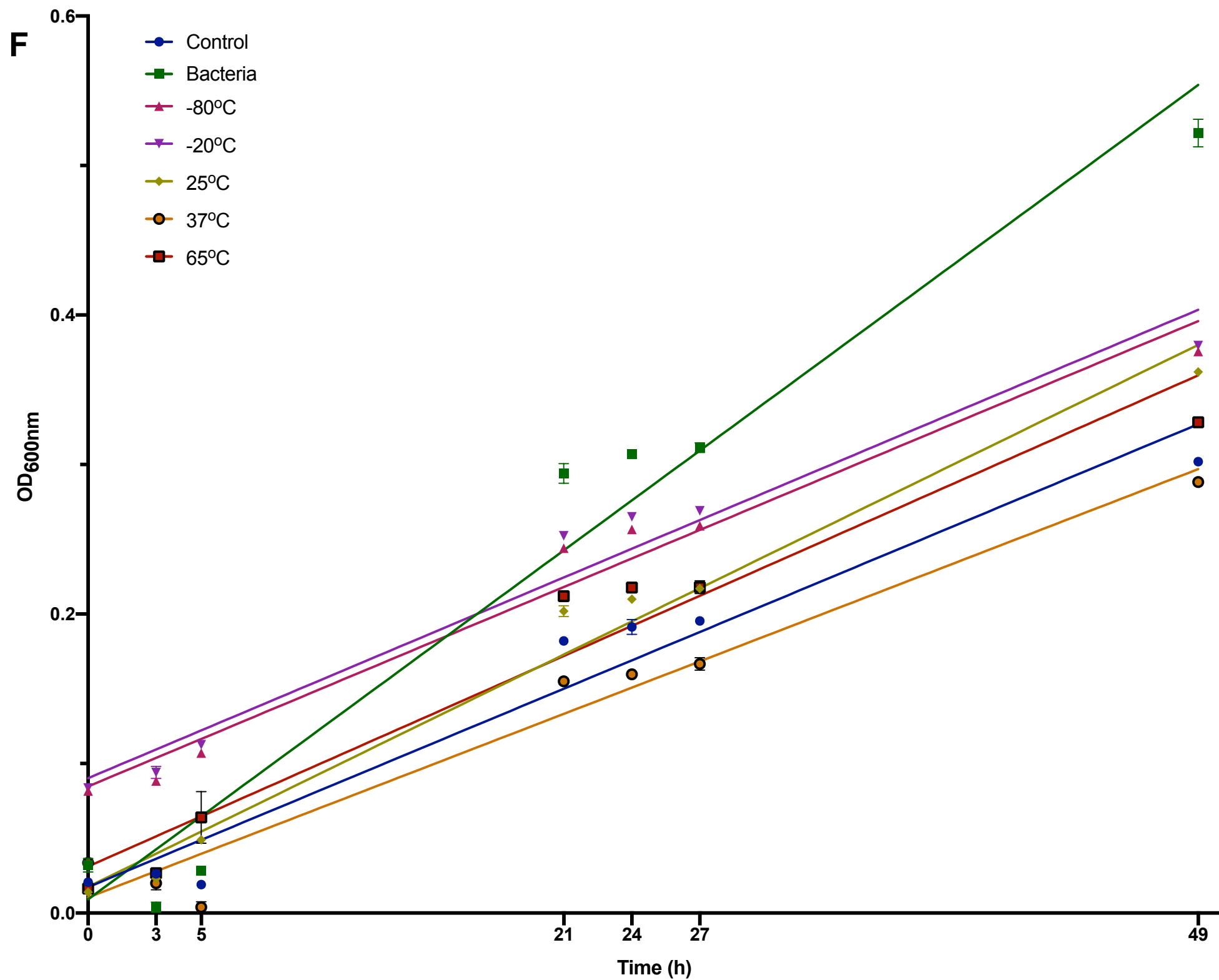

**G**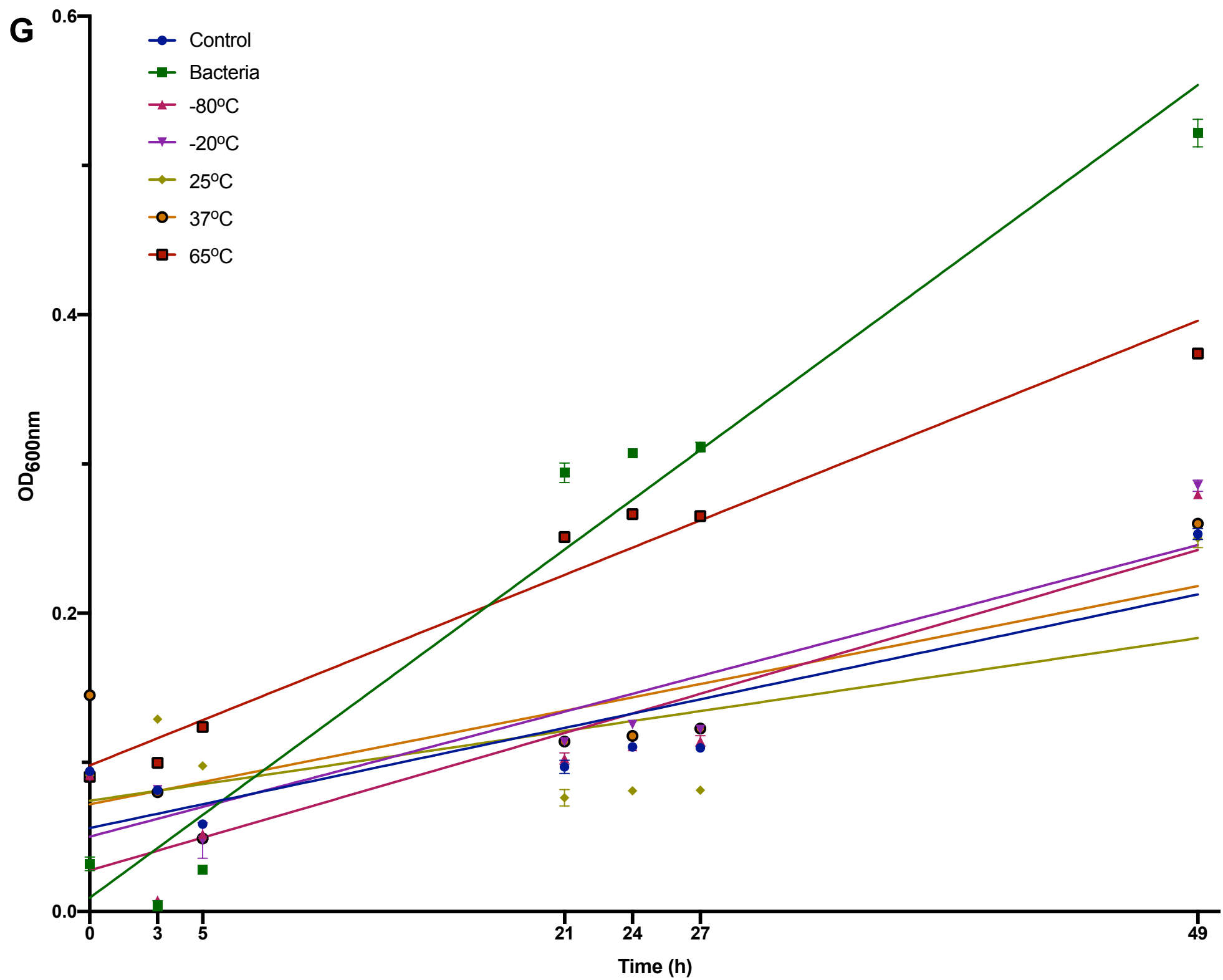

Supplement: S1 Fig — (A) Phage cocktail, (B) φMA1, (C) φMA1A, (D) φMA2, (E) φMA5, (F) φMA6, (G) φMA7. Only Pectobacterium mix (Bacteria) used as positive control and phage/phage cocktail as a control sample (Control). Bars indicated ± standard error. (PDF) [file pone.0230842.s004.pdf]

**A**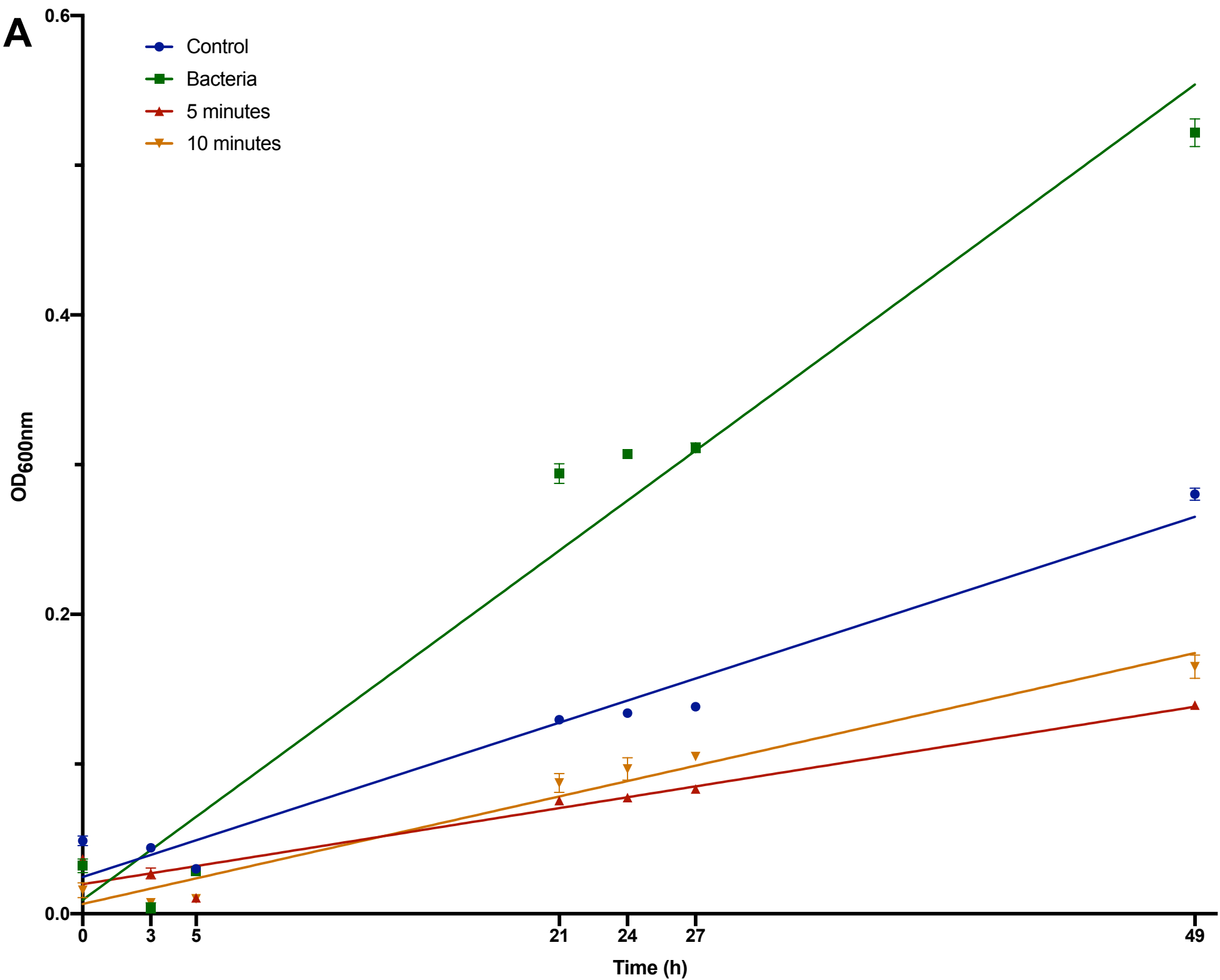

**B**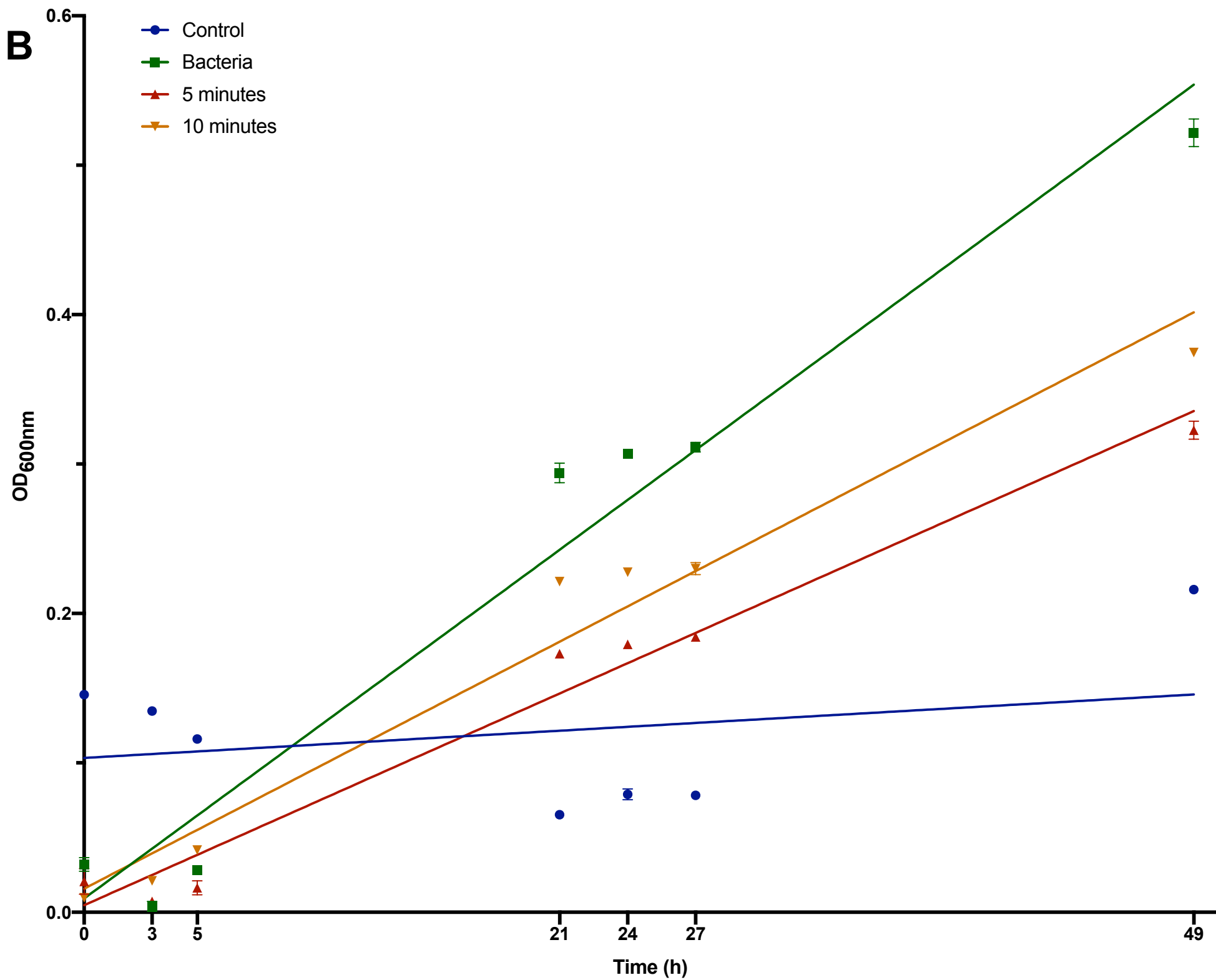

**C**

- Control
- Bacteria
- 5 minutes
- 10 minutes

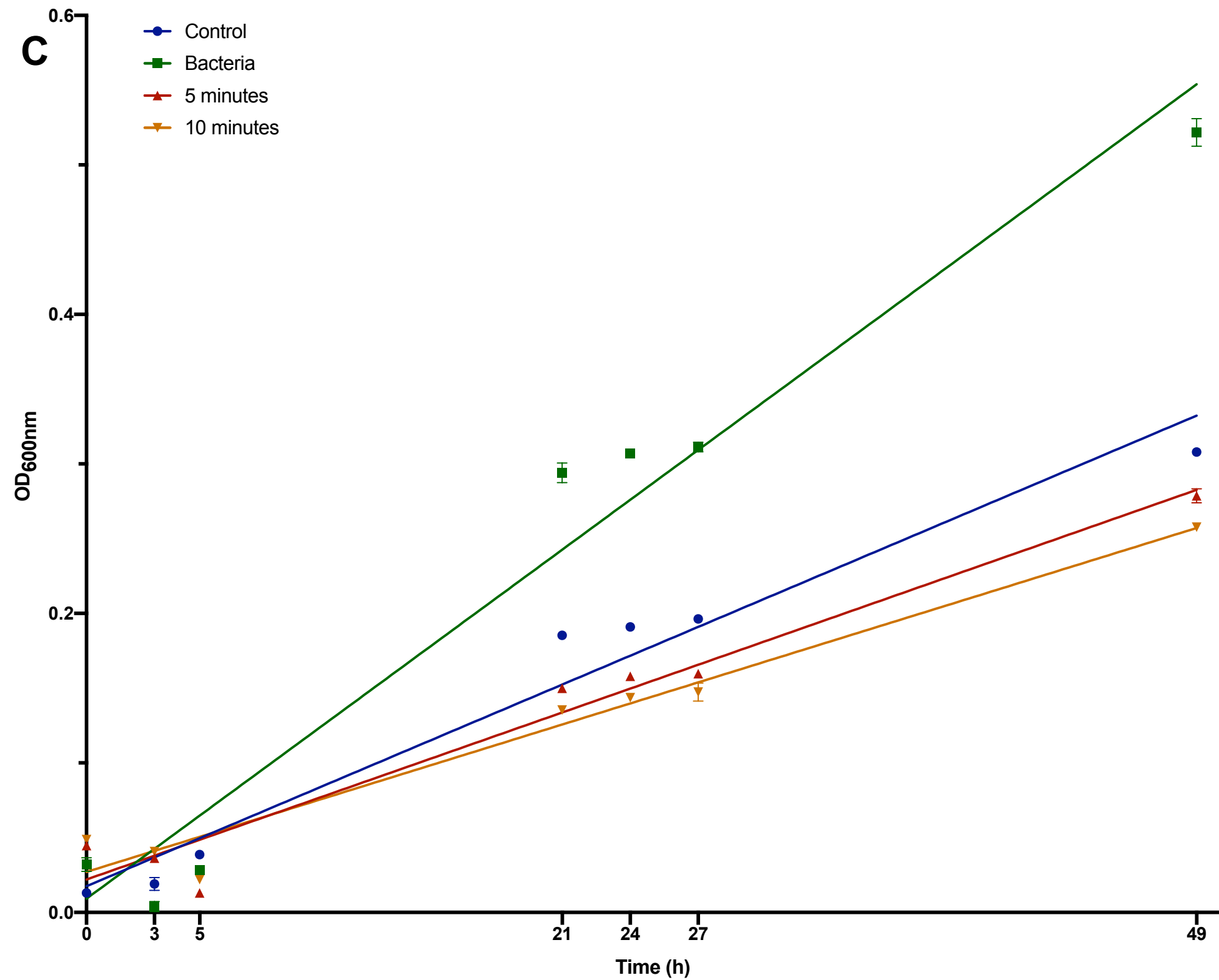

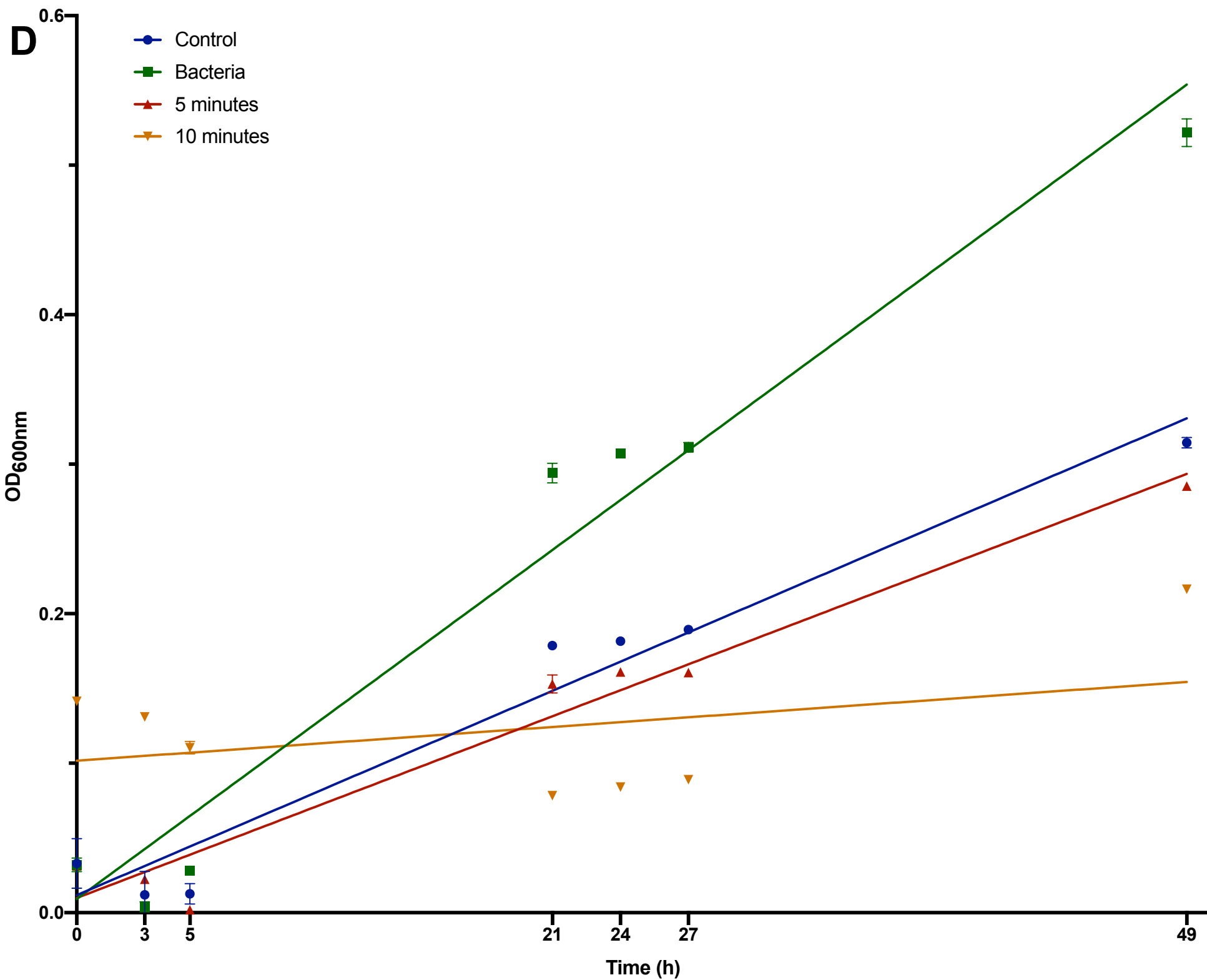

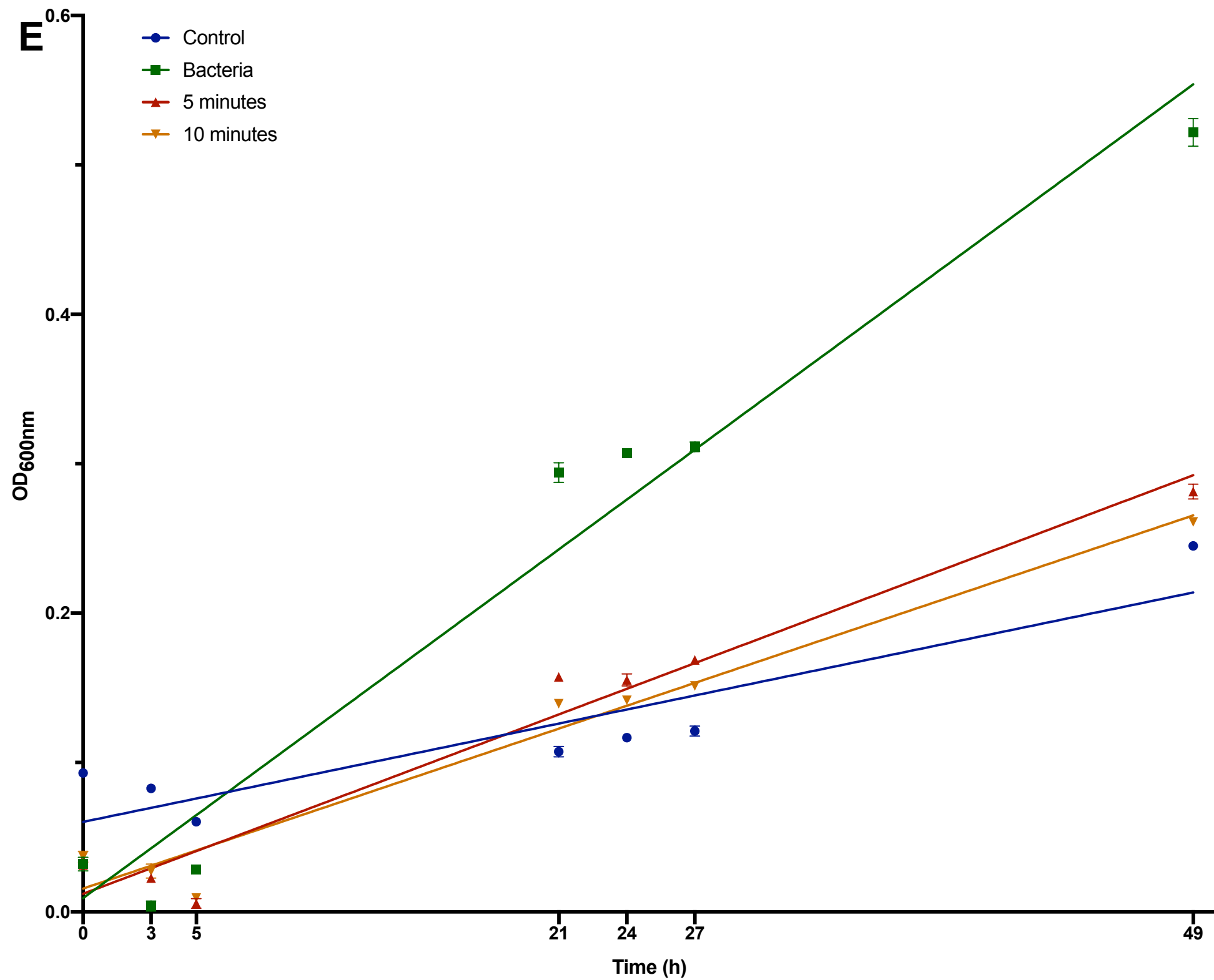

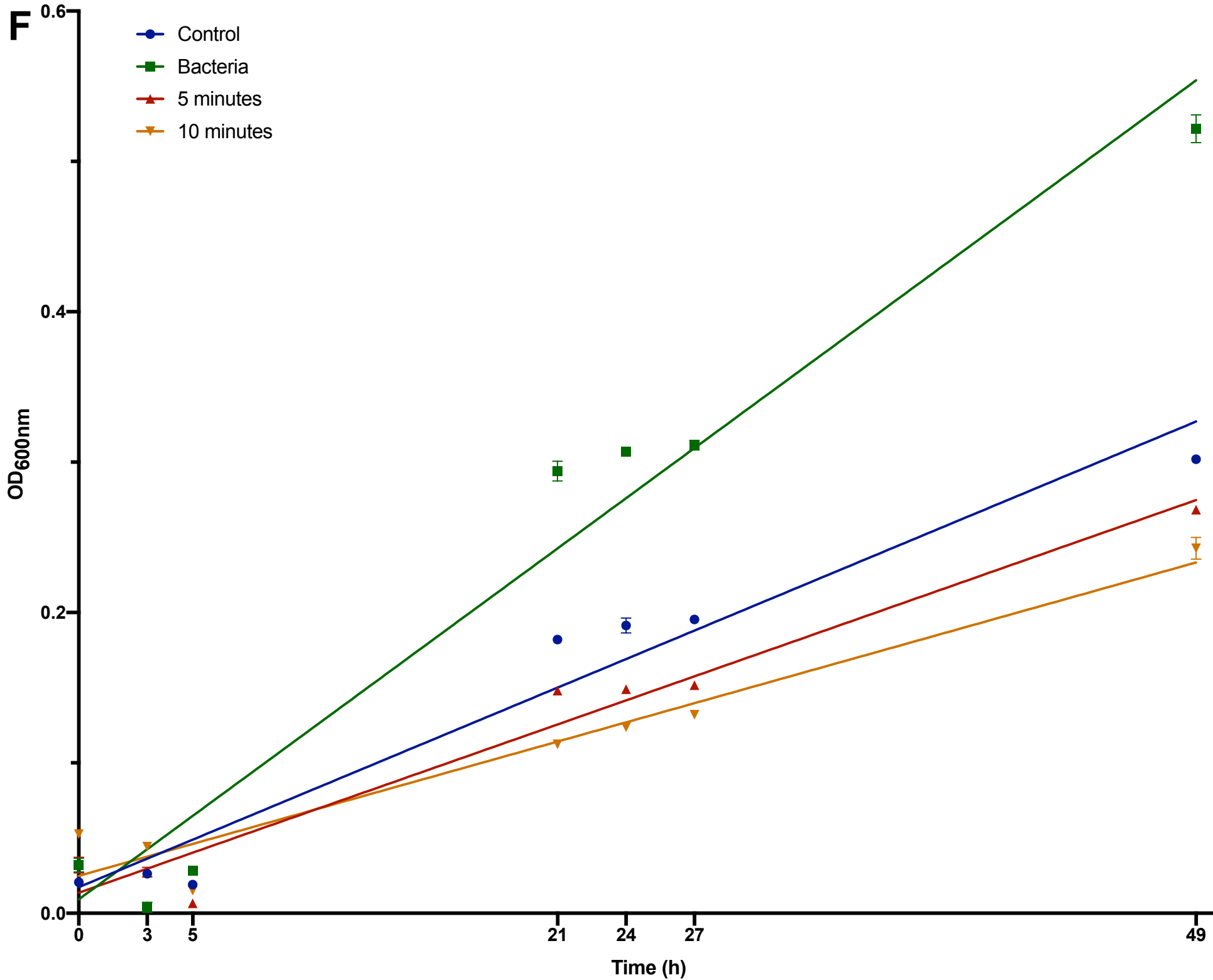

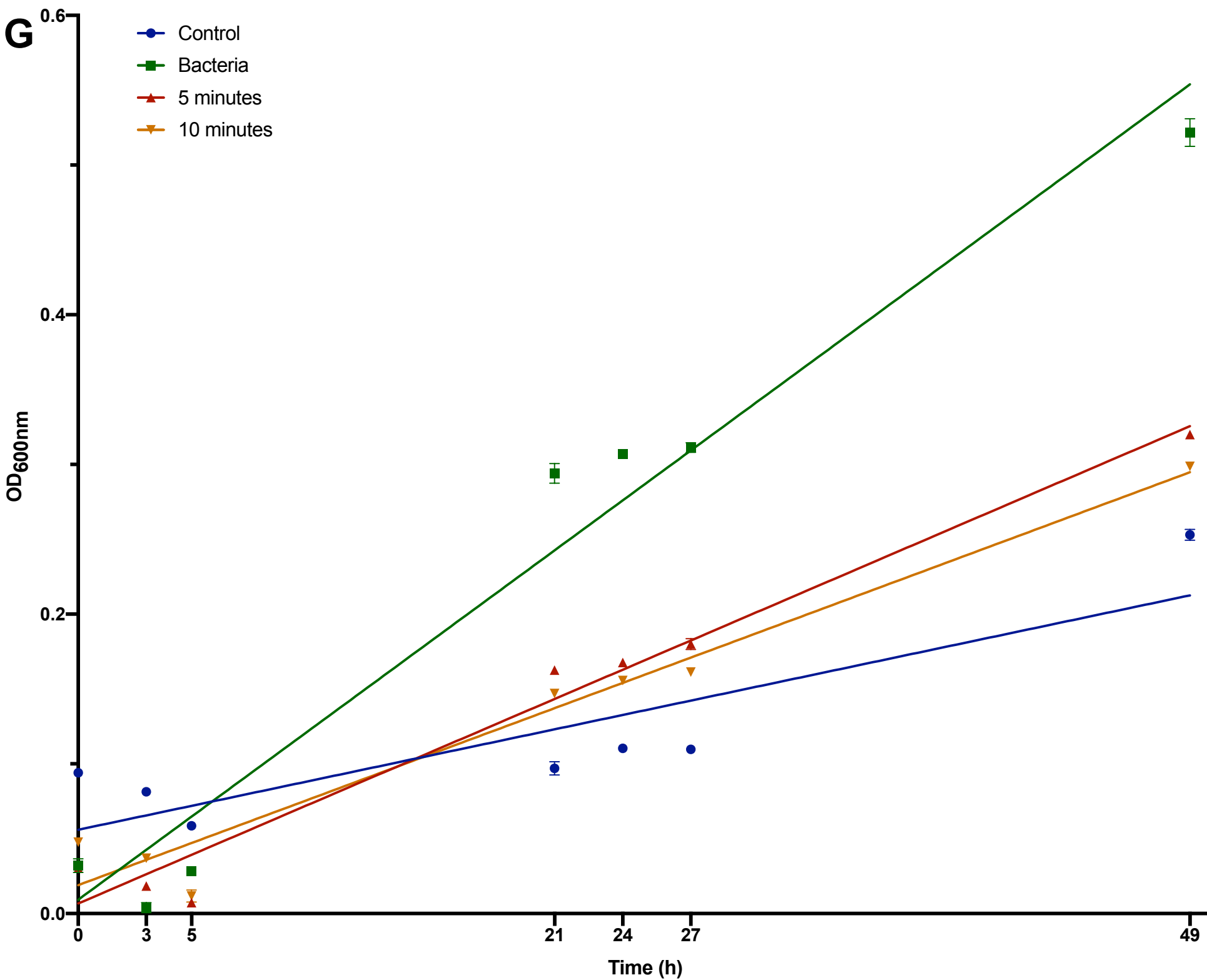

Supplement: S2 Fig — (A) Phage cocktail, (B) Phage φMA1; (C) Phage φMA1A; (D) Phage φMA2; (E) Phage φMA5; (F) Phage φMA6; (G) Phage φMA7. Only Pectobacterium mix (Bacteria) used as positive control and phage/ phage cocktail as a control sample (Control). Bars indicated ± standard error. (PDF) [file pone.0230842.s005.pdf]

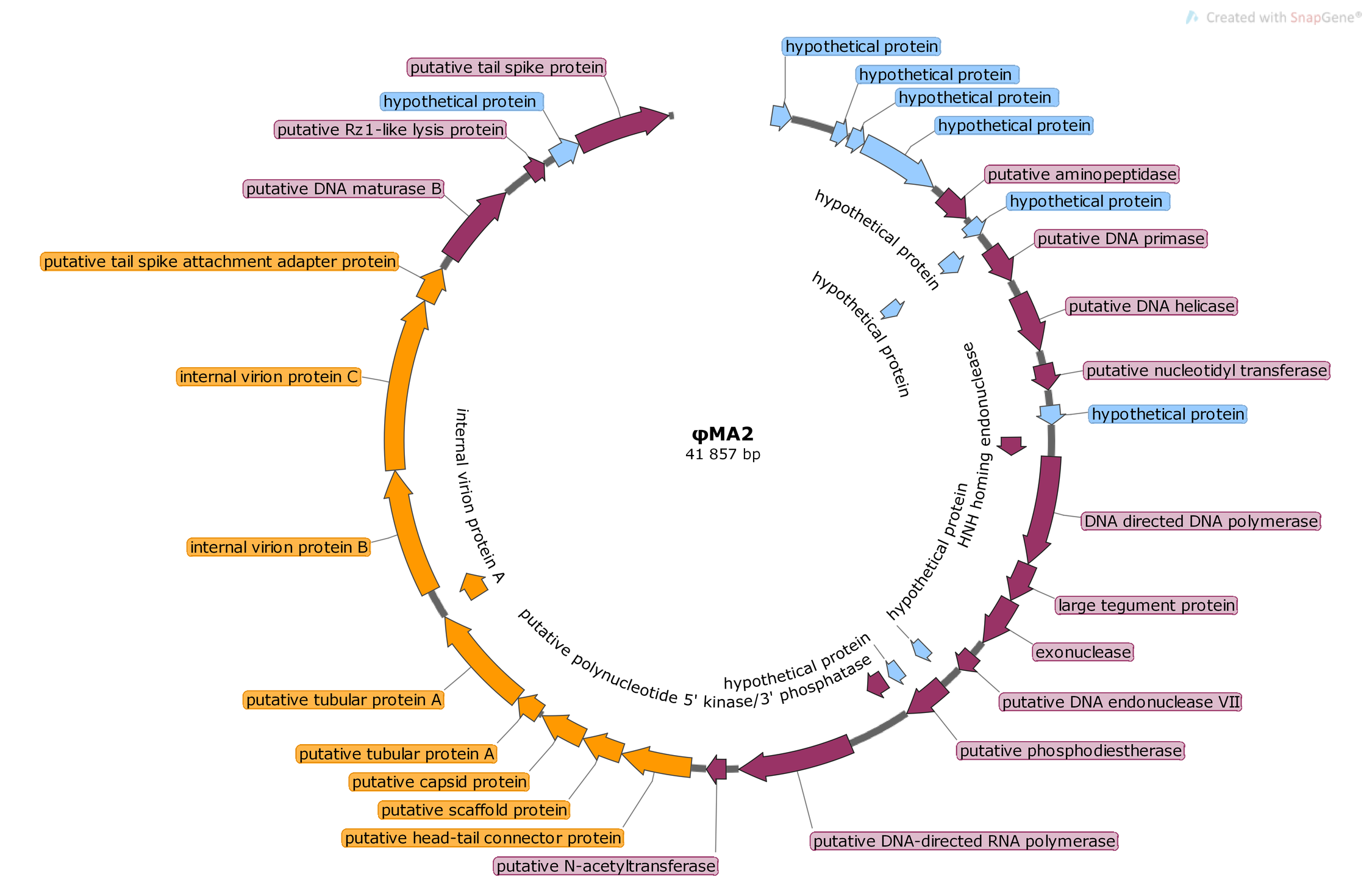

Supplement: S3 Fig — Different colours indicate coding for the following proteins: hypothetical (blue), structural proteins (orange), proteins for phage replication and lifecycle (pink). (TIF) [file pone.0230842.s006.tif]

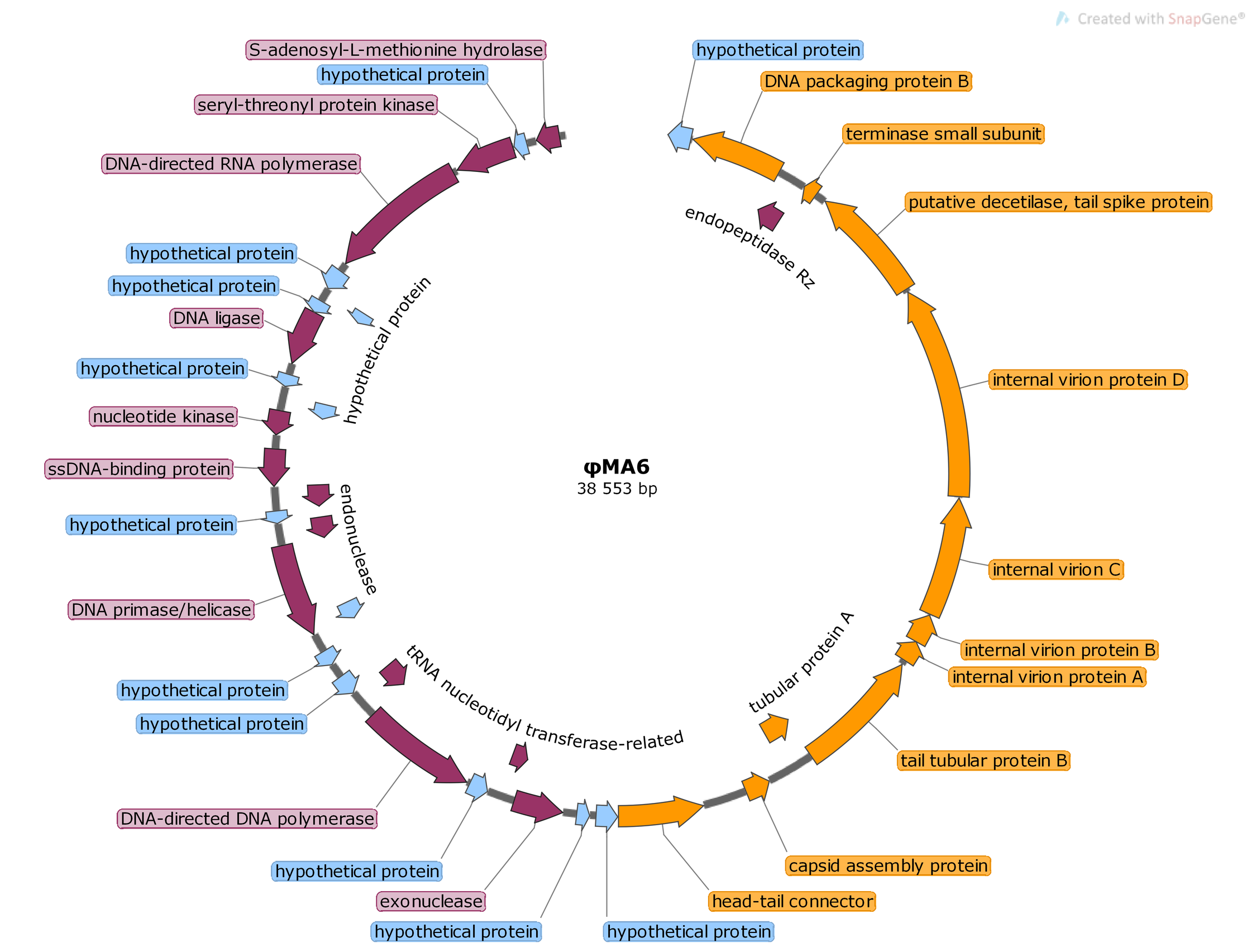

Supplement: S4 Fig — Different colours indicate coding for the following proteins: hypothetical (blue), structural proteins (orange), proteins for phage replication and lifecycle (pink) (TIF) [file pone.0230842.s007.tif]

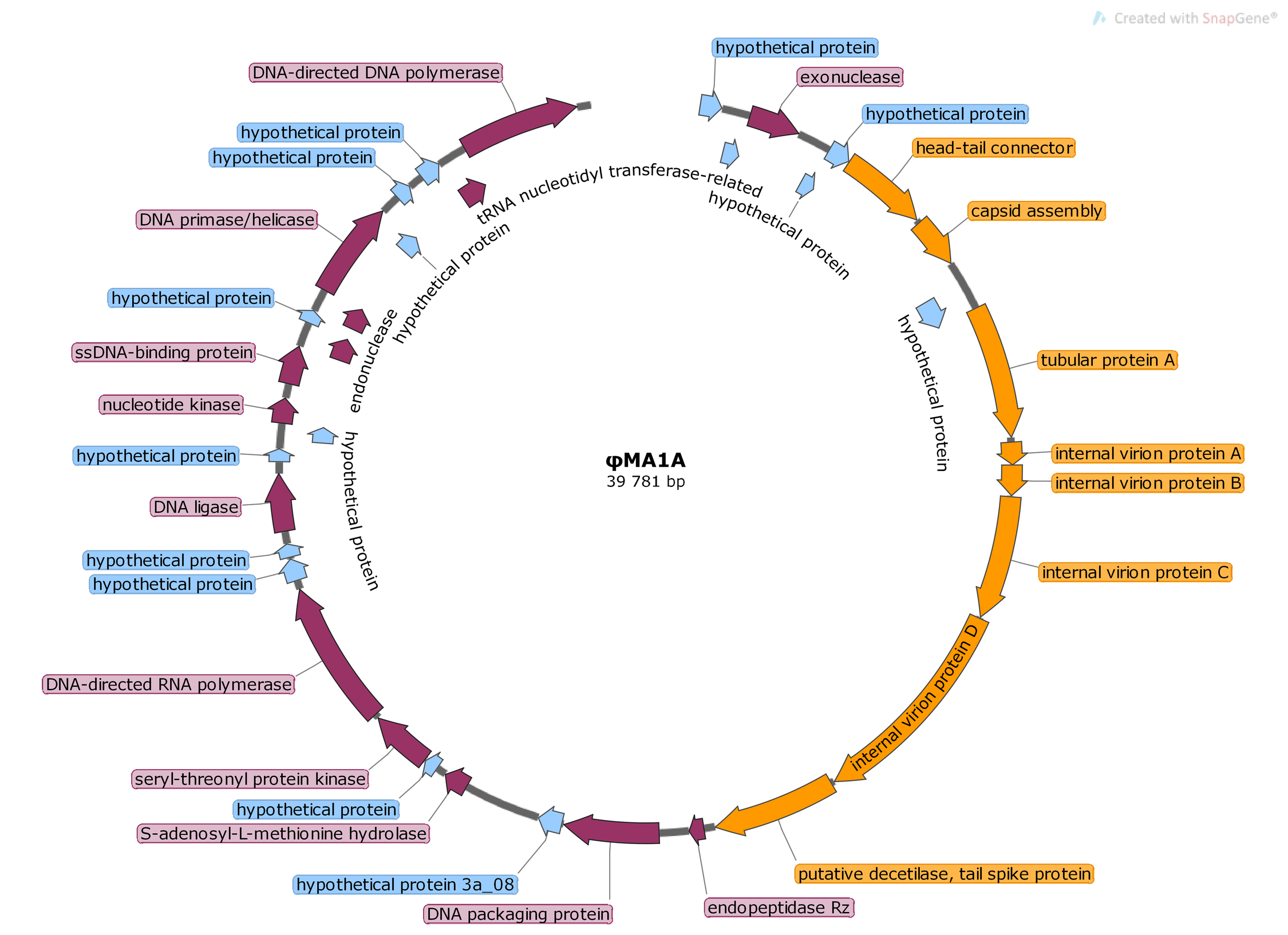

Supplement: S5 Fig — Different colours indicate coding for the following proteins: hypothetical (blue), structural proteins (orange), proteins for phage replication and lifecycle (pink). (TIF) [file pone.0230842.s008.tif]
